# The Adaptation of *Botrytis cinerea* Extracellular Vesicles Proteome to Surrounding Conditions: Revealing New Tools for Its Infection Process

**DOI:** 10.3390/jof9090872

**Published:** 2023-08-24

**Authors:** Almudena Escobar-Niño, Anne Harzen, Sara C. Stolze, Hirofumi Nakagami, Francisco J. Fernández-Acero

**Affiliations:** 1 Microbiology Laboratory, Institute for Viticulture and Agri-Food Research (IVAGRO), Faculty of Environmental and Marine Sciences, Department of Biomedicine, Biotechnology and Public Health, University of Cádiz, 11510 Puerto Real, Spain; franciscojavier.fernandez@gm.uca.es; 2 Protein Mass Spectrometry, Max Planck Institute for Plant Breeding Research, 50829 Cologne, Germany; harzen@mpipz.mpg.de (A.H.); stolze@mpipz.mpg.de (S.C.S.); nakagami@mpipz.mpg.de (H.N.); 3 Basic Immune System of Plants, Max Planck Institute for Plant Breeding Research, 50829 Cologne, Germany

**Keywords:** *Botrytis cinerea*, extracellular vesicles proteome, infection tool, virulence/pathogenicity factors, cell wall degrading enzyme, unconventional secretion

## Abstract

Extracellular vesicles (EVs) are membranous particles released by different organisms. EVs carry several sets of macromolecules implicated in cell communication. EVs have become a relevant topic in the study of pathogenic fungi due to their relationship with fungal–host interactions. One of the essential research areas in this field is the characterization protein profile of EVs since plant fungal pathogens rely heavily on secreted proteins to invade their hosts. However, EVs of *Botrytis cinerea* are little known, which is one of the most devastating phytopathogenic fungi. The present study has two main objectives: the characterization of *B. cinerea* EVs proteome changes under two pathogenic conditions and the description of their potential role during the infective process. All the experimental procedure was conducted in *B. cinerea* growing in a minimal salt medium supplemented with glucose as a constitutive stage and deproteinized tomato cell walls (TCW) as a virulence inductor. The isolation of EVs was performed by differential centrifugation, filtration, ultrafiltration, and sucrose cushion ultracentrifugation. EVs fractions were visualised by TEM using negative staining. Proteomic analysis of EVs cargo was addressed by LC-MS/MS. The methodology used allowed the correct isolation of *B. cinerea* EVs and the identification of a high number of EV proteins, including potential EV markers. The isolated EVs displayed differences in morphology under both assayed conditions. GO analysis of EV proteins showed enrichment in cell wall metabolism and proteolysis under TCW. KEGG analysis also showed the difference in EVs function under both conditions, highlighting the presence of potential virulence/pathogenic factors implicated in cell wall metabolism, among others. This work describes the first evidence of EVs protein cargo adaptation in *B. cinerea*, which seems to play an essential role in its infection process, sharing crucial functions with the conventional secretion pathways.

## 1. Introduction

Extracellular vesicles (EVs) are double-layer phospholipid membrane spheric particles that are released by all three domains of life cells to the extracellular environment [1,2]. EVs transport different cargo with a multitude of proteins, nucleic acids, and lipids that make them present heterogeneous biological functions. For example, EVs act in cell-to-cell communication and in multiple signaling pathways [1,2]. These structures have been reported to be essential in determining the plant–microbe interaction outcome by the modulation of the immune system and plant defense or the delivery of virulence-associated molecules [1,2]

EVs are heterogeneous in biogenesis pathways and size. Eukaryotic EVs are usually classified into three groups, apoptotic bodies, microvesicles, and exosomes [2]. Apoptotic bodies are vesicles formed during apoptosis, which are larger than 1000 nm. Microvesicles are medium vesicles, from 50 nm to 1000 nm, that are generated by the outward budding of the plasma membrane. Exosomes are small vesicles, ranging from 30 to 150 nm in diameter, which are released by the endosomal pathway during the maturation of multivesicular bodies (MVB) [2]

From 1973, when the first microscopical evidence of the presence of secreted vesicles in fungi was highlighted in *Cryptococcus neoformans* [3], EVs from twenty different species of yeast and filamentous fungi, which comprise pathogenic and nonpathogenic species have been described [2].

Such as EVs are a relevant mechanism of virulence-factors secretion; it has been studied in many emerging and opportunistic fungal clinically important pathogens from the genera *Candida, Cryptococcus, Histoplasma, Aspergillus, Sporothrix, Paracoccidiodes, Malassezia, Rhizopus, Exophiala, Pichia*, *Trichophyton* and *Talaromyces* [2,4]. In the area of phytopathology, EVs production was reported in many species such as *Alternaria infectoria*, *Trichoderma reesei*, *Fusarium oxysporum* f. sp. *vasinfectum*, *Fusarium graminearum* [5], *Zymoseptoria tritici*, *Penicillium digitatum*, *Colletotrichum higginsianum* [2,6], and recently in *Botrytis cinerea* [7].

*B. cinerea* is one of the most devastating phytopathogenic fungi, being the causal agent of the plant disease gray mold [8]. Its attack on hundreds of essential agronomic crops generates huge economic losses for farmers around the world [8]. Since the *B. cinerea* genome was published [9,10], molecular approaches to *B. cinerea* have been facilitated. Most of these studies were focused on determining the role of virulence or pathogenicity factors of specific genes. This molecular information has been collected in the “pathogen–host interaction database” [11]. From this list, it seems clear that most of the detected genes with crucial roles in pathogeny/virulence are related to the signaling cascades. To collect as much information as possible on the proteins involved in the *B. cinerea* signaling machinery, several proteomics approaches have been developed using two different carbon sources, glucose as a constitutive stage and deproteinized tomato cell walls (TCW) as virulence inductor [12]. These assayed conditions have shown specific fungal responses in biological process essentials in the infective cycle, such as a toxin or cell wall degrading enzyme production [13]. One of the most important research areas in the analysis of EVs is the characterization of EVs cargo, which includes numerous non-randomly packed proteins. As plant–host interaction, EVs protein cargo, and its release are influenced by the environmental conditions [4]. So, unraveling the protein composition of EVs from phytopathogenic fungi under different conditions could help us to understand more about its infective cycle. Proteomic profiling of EVs cargo has presented a challenge due to the variability of developed isolation protocols [14]. Mass spectrometry (MS) techniques were implemented as a powerful tool for the identification and quantification of proteins. Methodology and instrumental improvements have increased the level of detection, making it more sensitive and applicable to the analysis of such difficult proteomes [2,15].

In this paper, the proteome of *B. cinerea* EVs has been analyzed to unravel key points in the infection process regulation, crucial to overcome plant defenses. In addition, this work points out new potential virulence/pathogenicity factors that could be used as therapeutical targets in the control of gray mold rot disease. The obtained results have highlighted *B. cinerea* EVs as an essential tool in the infective process, working together with the conventionally secreted proteins. The analysis of this proteome has shown their implication in crucial steps of plant–pathogen interaction, such as the release of cell wall degrading and modification enzymes.

## 2. Materials and Methods

### 2.1. Fungal Strains and Culture Conditions

The fungal strain *B. cinerea* B05.10 was provided by the Spanish Type Culture Collection (CECT). Preparation and maintenance of conidial stock suspensions were performed as previously reported [16]. Briefly, cultures were grown in Petri dishes containing maltagar (MA) (2% malt extract, 2% agar, (%, *w*/*v*)) and maintained in incubators under alternating (12 h) light/dark cycles at 21 °C. For storage, conidia were harvested in 0.01% (*v*/*v*) Tween 20 solution, filtered through a 30 µm nylon filter (Sefar Nytal, Mays, Barcelona), and centrifuged for 5 min at 120× *g*. The resulting conidial pellet was suspended to a concentration of 10^7^ conidia/mL in a 10% (*v*/*v*) glycerol solution for its storage at −80 °C or in NaCl 0.9% for its direct use [17]. Culture media were prepared using two different carbon sources: glucose (GLU) (Panreac, Barcelona, Spain) as the constitutive stage; and deproteinized tomato cell walls (TCW) as the virulence inductor, as previously described [16]. Briefly, lyophilized tomatoes were grinded to a fine powder in liquid nitrogen using a mortar and pestle. The frozen powder was then washed five times for 5 min with 2.5 volumes of cold 0.1 M potassium phosphate buffer (pH 7). This insoluble material was then washed with NaCl (1 M), distilled water, chloroform–methanol (1:1) (five times), cold acetone (three times), and ethyl acetate. The remaining insoluble residue, which constitutes the deproteinized cell walls, was air-dried and stored at 4 °C [18].

One liter flask containing 400 mL of minimal salt medium (MSM) (50 mM NH_4_Cl, 7.3 mM KH_2_PO_4_, 4.2 mM MgSO_4_, 6.7 mM KCl, 0.07 mM FeSO_4_) supplemented with 1% of each carbon source was inoculated with fresh 5 × 10^4^ conidia/mL of *B. cinerea*. After 5 days of culture at 180 rpm at 22 °C under alternating 12-h light/dark cycles, fungal mycelium was harvested by filtration with a 30 μm nylon filter (Sefar Nytal, Heiden, Switzerland). Three biological replicates of each culture condition were incubated in parallel.

### 2.2. Optimizations of EVs Isolation

For the optimization of the EVs isolation, two previously reported protocols were used as references. These protocols were the reported ones for the fungi *Zymoseptoria tritici* [19] and *Fusarium oxysporum* [20] with some modifications [21]. Briefly, 400 mL of *B. cinerea* 5-day-old cultures were supplemented with 100 µL of cOmplete™, Mini, EDTA-free Protease Inhibitor Cocktail (Roche) dissolved in 1 mL of 1× phosphate buffer, pH 7.0. *B. cinerea* mycelium was removed from the culture medium by filtration through a sterile 30 μm nylon filter (Sefar Nytal, Heiden, Switzerland). Cells debris and spores were removed from broth cultures by centrifugations at 4500× *g* (4 °C) for 25 min and at 15,000× *g* (4 °C) for 45 min. Supernatant was carefully removed and passed through a 0.45 μm membrane filter (Thermo Scientific™, Waltham, MA, USA) using vacuum filtration. Then, supernatants were concentrated using an ultrafiltration filter Pierce™ Protein Concentrator PES, 100K MWCO, 5–20 mL (cutoff filter 100 kDa) (Thermo Scientific™, Waltham, MA, USA) by centrifugation at 6000× *g* for 30 min. About 30 mL of Vesicle-enriched supernatant was loaded slowly over 4 mL of 30% sucrose solution, prepared in 1× PBS (0.137 M NaCl, 0.0027 M KCl, 0.01 M Na_2_HPO_4_, 0.0018 M KH_2_PO_4_) (PBS was filter sterilized with 0.22 μm membrane), forming a layer, and centrifuged at 100,000× *g*, 4 °C for 90 min in open-top Ultra Clear 38.5 mL tubes (Beckman Coulter, Indianapolis, IN, USA) using an SW 32 Ti swinging bucket rotor and Optima XPN-100 ultracentrifuge (Beckman Coulter, Indianapolis, IN, USA) (Centrifugation and Ultracentrifugation Unit-INBIO, University of Cadiz). The supernatant and the sucrose layer (~5 mL) were separated. On the one hand, supernatant was ultracentrifuged for 80 min at 100,000× *g* and 4 °C to pellet down remaining EVs (supernatant of this ultracentrifugation step will be used as control/supernatant control). This EVs pellet diluted with PBS was loaded onto the previously obtained sucrose layer and ultracentrifuged for 90 min at 100,000× *g* and 4 °C to pellet down EVs. Supernatant was removed completely, and the EVs containing sucrose layer were diluted and washed with 1× PBS, pH 7.4, sterilized by ultracentrifugation at 100,000× *g*, 90 min, and 4 °C. The EVs pellet was washed with 1× PBS, pH 7.4, by ultracentrifugation at 100,000× *g*, 90 min, and 4 °C. Finally, the EVs pellet was resuspended in 1 mL sterile 1× PBS (pH 7.4). Protein content was measured using the Qubit™ protein assay kit (Invitrogen, Waltham, MA, USA) in a Qubit^®^ 2.0 fluorometer (Invitrogen, Waltham, MA, USA) following the manufacturer’s instructions. Finally, EV samples were stored at −80 °C.

The optimized protocol was repeated with MSM supplemented with 1% of axenic TCW as a control (uninoculated control) in order to confirm that EV-like particles were not an artifact of the culture medium.

### 2.3. Transmission Electron Microscopy

EVs samples (15 µL) were deposited onto carbon-coated 200 mesh copper grids (AGS160H, Agar Scientific, Essex, UK) that had been previously washed in acetone and incubated for 7 min. Excess sample was blotted from grids with Whatman^®^ grade 1 qualitative filter paper (GE Healthcare, Chicago, IL, USA). Samples were stained with 10 µL of 2% (*v*/*v*) uranyl acetate (UA) (Agar Scientific, Essex, UK) onto grids for 3 min. The excess stain solution was blotted off, and the grids were dried overnight. Images were captured using a TALOS electron microscope (Talos F200X) (Thermo Scientific, Waltham, MA, USA) operated at 200 kV (UCA Central Services). The images obtained were used to calculate the concentration of vesicles (EV/1 µm^2^) and the average size of the vesicles (nm).

### 2.4. Protein Extraction

Three different fractions were subjected to protein extractions. Firstly, to identify proteins present inside the EV, the vesicles were sonicated twice for 30 s with UP50H Compact Lab Homogenizer (amplitude 80%, cycle 0.5, 50 watts, 30 kHz; Hielscher Ultrasonics, Teltow, Germany) after adding 1% of SDS. Proteins from vesicle suspensions were then precipitated by adding 6 volumes of ice-cold acetone and incubating overnight at −80 °C. Proteins present in the supernatant of ultracentrifugation steps used as a control (supernatant control) were precipitated by adding 6 volumes of ice-cold acetone and incubating overnight at −80 °C. Finally, proteins from mycelia were extracted using CK14 7 mL homogenizing kit (Precellys^®^ 7 mL Soft Tissue Homogenizing Kit) by grinding 0.3 g mycelia in 6 mL of PBS +1% of SDS, with ceramic beads (1.4 mm zirconium oxide beads, Precellys; Bertin Technologies, Montigny-le-Bretonneux, France) using a Minilys (Bertin Technologies, Montigny-le-Bretonneux, France) at maximum level, 5 cycles of 30 s. Lysates were clarified by centrifugation at 10,000× *g* for 10 min at 4 °C, retaining the supernatant. Proteins were precipitated by adding 6 volumes of ice-cold acetone and incubating overnight at −80 °C. Proteins were precipitated by centrifugation at 10,000× *g* for 15 min at 4 °C in each sample fraction (EVs, supernatant control, and mycelium). Protein digestion was performed using FASP small-scale protocol. To this aim, the protein pellet was dissolved in denaturing buffer (8 M Urea, 100 mM Tris-HCl pH 8.5, 5 mM DTT) and incubated for 30 min at room temperature for 30 min to allow protein reduction. Then, protein concentration was determined using Pierce 660 protein assay reagent (Thermo Scientific™, Waltham, MA, USA) following the manufacturer’s instructions. 450 µL of 0.111 µg/µL of protein aliquot diluted in buffer UA (8 M Urea, 100 mM Tris-HCl pH 8.5) was added to a 30 kDa cutoff filter unit (Vivacon 500, VN01H22; Sartorius, Göttingen, Germany) and centrifuged at 14,000× *g* for 10 min. Then, the filter was washed with 450 µL of buffer UA at 14,000× *g* for 10 min. Alkylation of protein was performed by adding 100 µL of CAA solution (55 mM chloroacetamide, 8 M Urea, 100 mM Tris-HCl pH 8.5) and incubating at room temperature for 30 min in the dark. After centrifuging the filter at 14,000× *g* for 10 min and washing it twice with 450 µL of buffer UA, 450 µL of dilution solution (100 mM Tris-HCl pH 8.5, 1 mM CaCl_2_) was added. Protein digestion was performed with 1:100 trypsin (Pierce Trypsin MS-Grade, 90057, Thermo Scientific, Waltham, MA, USA) to protein ratio at 37 °C overnight. Peptides were collected by centrifuging the unit filter at 14,000× *g* for 10 min and acidified with 20% of TFA (Trifluoroacetic acid) solution (final concentration 0.5%). Peptide solution was filtered using C18 matrix packed in yellow pipette tips as described by Rappsilber et al. [22]. C18 matrix was equilibrated with 75 μL Methanol, 75 μL Solution B (80% acetonitrile, 0.5% FA), and 75 μL Solution A (2% acetonitrile, 0.1% TFA) by centrifugation at 1200× *g*, 3 min. Peptide solution (max. 50 μg) was loaded by centrifugation at 800× *g* for 5–10 min. The tip was washed with 75 μL solution A at 1200× *g*, 3 min.). Peptides were eluted from the matrix with 25 μL solution B by centrifugation at 500× *g* for 5 min. The elution step was repeated once more. Eluted peptide was dry in a Vacuum Concentrator (Vacufuge Vacuum Concentrator 5301 Centrifuge; Eppendorf, Hamburg, Germany) for 40 min. Peptide pellet was resuspended in 10μL buffer A* (2% acetonitrile, 0.1% TFA) and peptide concentration was measured in Nanodrop 2000 (Thermo Scientific™, Waltham, MA, USA). Peptide solution was diluted to 0.1 μg/μL for LC-MS analysis.

### 2.5. Proteomic Analysis by LC-MS Analysis

Dried peptides were re-dissolved in 2% the, 0.1% TFA (10 µL) and diluted to 0.1 µg/µL for analysis. Samples were analyzed using an EASY-nLC 1200 (Thermo Fisher) coupled to a Q Exactive Plus mass spectrometer (Thermo Fisher). Peptides were separated on 16 cm frit-less silica emitters (New Objective, 75 µm inner diameter), packed in-house with reversed-phase ReproSil-Pur C18 AQ 1.9 µm resin (Dr. Maisch). Peptides were loaded on the column and eluted for 115 min using a segmented linear gradient of 5% to 95% solvent B (0–5 min: 5% of B; 5–65 min from 5% to 20% of B; 65–90 min: from 20% to 35% of B; 90–100 min: from 35% to 55% of B; 100–105 min: from 55% to 95% of B; 105–115 min: 95% of B) (solvent A 0% ACN, 0.1% FA; solvent B 80% ACN, 0.1%FA) at a flow rate of 300 nL/min. Mass spectra were acquired in data-dependent acquisition mode with a TOP15 method. MS spectra were acquired in the Orbitrap analyzer with a mass range of 300–1750 *m*/*z* at a resolution of 70,000 FWHM and a target value of 3 × 10^6^ ions. Precursors were selected with an isolation window of 1.3 *m*/*z*. HCD fragmentation was performed at a normalized collision energy of 25. MS/MS spectra were acquired with a target value of 10^5^ ions at a resolution of 17,500 FWHM, a maximum injection time (max.) of 55 ms, and a fixed first mass of *m*/*z* 100. Peptides with a charge of +1, greater than 6, or with an unassigned charge state were excluded from fragmentation for MS^2^. Dynamic exclusion for 30 s prevented repeated selection of precursors.

Raw data were processed using MaxQuant software (version 1.6.3.4), [23] with label-free quantification (LFQ) and iBAQ enabled [24]. MS/MS spectra were searched by the Andromeda search engine against a combined database containing the sequences of *B. cinerea* B05.10 proteome available at UniProt (UP000001798, downloaded 19 July 2022) and sequences of 248 common contaminant proteins and decoy sequences. Trypsin specificity was required, and a maximum of two missed cleavages were allowed. The minimal peptide length was set to seven amino acids. Carbami-domethylation of cysteine residues was set as fixed, oxidation of methionine and protein N-terminal acetylation as variable modifications. Peptide-spectrum-matches and proteins were retained if they were below a false discovery rate of 1%.

Comprehensive analysis of generated protein list was performed using the software Perseus 2.0.6.0 [25]. Identified proteins were filtered by removing contaminant proteins, proteins only identified by site or reverse sequence, proteins with less than two matching peptides, or those that were not present in at least two biological replicates of one fraction. LFQ intensities were log_2_ transformed, and significance was determined using a two-sample *t*-test with a permutation-based FDR for q-value calculation. *p*-value 0.01 and q-value 0.01 were used as cut-offs for the analysis. Proteins identified in all replicates of at least 1 group presenting log_2_-fold change (Log_2_-FC) values greater than 1 (FC 2) were considered upregulated, and all values log_2_-FC less than −1 (or FC = 0.5) were considered down regulated. Proteins exclusive of one condition (culture condition and/or fraction) were those presented in all replicates of one condition and in any replicates of the other condition. Common non-regulated proteins were those identified in all the replicates of two conditions, presenting no significant differences (*p*-value and/or q-value > 0.01 or 0.5 < FC < 2).

The mass spectrometry proteomics data have been deposited in the ProteomeXchange Consortium via the PRIDE partner repository [26], with the dataset identifier PXD040614 (Data accession: Username: reviewer_pxd040614@ebi.ac.uk; Password: TYslchG9).

### 2.6. Bioinformatic Analysis

Gene ontology (GO) and KEGG tools were used to perform functional analysis. The GO annotation of the reference proteome of *B. cinerea* B05.10 from Uniprot was extended with existing GO annotations and GO annotations based on sequence homology using GORetriever and Goanna tools from AgBase [27]. GA2GEO tool from AgBase was used to convert GORetriever and Goanna files between the gene association format and an NCBI GEO (Gene Expression Omnibus) type format. The GEO-type format was added to Uniprot GO annotations. Finally, GOSlimViewer was used to provide a high-level summary of functions for this dataset. The resulting list was used as the background to perform GO enrichment analysis using FunRich 3.1.3 custom database option [28]. Fold change analysis and Venn diagrams between each dataset were performed using Fold tool and Venn tool from FunRich 3.1.3. BlastKOALA [29] annotation tool version 2.2 and KoFAMKOala [30] were used for K number assignment to identify proteins. KEGG reconstruct pathway was used to “reconstruct” KEGG pathway maps and other network entities from the set of assigned K numbers [31].

Exclusive or overrepresented proteins of EVs fraction were further analysed using Conserved Domain (CD) search tool from NCBI [32], using the default parameters of Batch of Protein Sequences. For uncharacterized proteins without results in the CD search, Blastp was used to obtain as much available information as possible.

Global characterization of identified proteins was performed using several software. The software DeepTMHMM 1.0.13 was used for the prediction of alpha and beta transmembrane proteins [33]. To study other kinds of protein–membrane associations, the analysis of GPI association (NetG–I-1.1) [34] and protein lipidation (GPS-Lipid v1.0) [35] were performed. OutCyte 1.0 server [36] and DeepTMHMM 1.0.13 [33] were used to check protein role as secreted through classical or non-classical pathways. DeepLoc 1.0 was used for predicting protein subcellular localization [37]. Finally, prediction of apoplastic and cytoplasmic effectors in *B. cinerea* was performed using EffectorP 3.0 [38].

## 3. Results and Discussion

### 3.1. EVs Isolated from B. cinerea Showed Different Morphologies Depending on Used Plant-Based Elicitor

**Figure 1:**
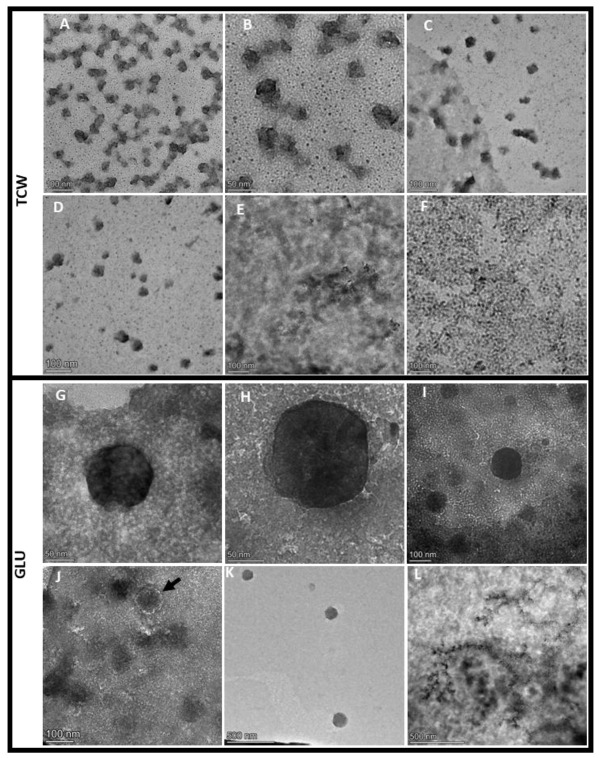
Morphology of *B. cinerea* EVs. TEM of EVs obtained from *B. cinerea* grown for 5 days in MSM liquid medium supplemented with TCW (**A**–**D**) or GLU (**G**–**K**) and their negative controls (**E**,**F**,**L**) (see Materials and Methods). Three controls are showed (**E**) supernatant control under TCW (supernatant from the ultracentrifugation step under TCW condition); (**F**) uninoculated control under TCW (MSM medium supplemented with TCW and without the fungus); and (**L**) supernatant control under GLU (supernatant from the ultracentrifugation step under GLU condition). Three distinct morphologies were found: (**A**–**D**) heavily pigmented vesicles with irregular morphology, (**G**,**H**,**J**,**K**) heavily pigmented vesicles with spheric morphology; and (**I**) heavily pigmented vesicles with multi-lobed rosette morphology (black arrow). Scale bar ranging from 50 nm to 500 nm.

To ascertain whether *B. cinerea* B05.10 released vesicles under different culture conditions, as reported in other fungi, we grew it for 5 days in MSM supplemented with GLU or TCW. EVs were purified from culture supernatants of the fungus by differential centrifugation and ultracentrifugation combined with filtration, ultrafiltration, and sucrose cushion ultracentrifugation, which was used to increase EVs purity by avoiding protein contamination [21]. EVs fractions were analysed by TEM using negative staining with UA. TEM analysis showed typical EV structures previously reported in other fungi [20,39], such as a sphere, multi-lobed and irregular vesicles (Figure 1A,B,E,F and Supplementary Figure S1). Moreover, EV-like structures were not revealed in control fractions (Figure 1C,D,H). Comparative TEM analysis between conditions demonstrated that they were diverse in size and shape. This comparison showed a higher amount of spherical EVs (Figure 1E,F) and the presence of multi-lobed rosette morphology (Figure 1G) under GLU conditions. On the contrary, under TCW, most EVs showed an irregular structure (Figure 1A,B). In addition, *B. cinerea* produced 93.18 EV/1 µm^2^ (st dev 75.06) under TCW condition and 13.87 EV/1 µm^2^ (st dev 5.43) under GLU, with a particle diameter of 30–45 nm and 30–130 nm, respectively. It is well reported that uranyl acetate dehydration changes the size of EVs, so to properly measure EVs’ size and concentration, further Nanoparticle tracking analysis (NTA) must be performed. Under both conditions, the majority of EVs were heavily pigmented vesicles, such as those reported for *Alternaria infectoria* [39]. Pigmented vesicles could also be generated by the sucrose cushion ultracentrifugation step of the isolation method [21]. GLU results agree with previously reported EVs isolated from the supernatant of *B. cinerea* growing in a minimum medium supplemented with 2% of glucose, which described spheroid vesicles of 30–400 nm [7]. However, the same authors described the isolation of ovoid and tubular vesicles of 50 to 500 nm in size from *B. cinerea* grown on a solid medium with cellophane [7]. Altogether, these differences observed under several culture conditions indicate that the culture medium strongly affects the production of EVs and probably their function/cargo. Similar results have been previously reported for *C. neoformans* EVs [40] and *Histoplasma capsulatum* [41], among others. In addition, these differences in EV production agree with previous results in *B. cinerea*, showing phenotypic changes of the fungus under both assayed conditions [13].

Finally, the average protein concentration of the EVs fraction measured by Qubit was 0.17 μg/mL of initial culture (st. dev. 0.01) in GLU and 0.1975 μg/mL of initial culture (st. dev. 0.04) in TCW. These results for the protein content of the *Botrytis* vesicles are in agreement with those described for other fungi such as *Fusarium oxysporum* [20]. The observed morphological differences [13] must be ascribed to the nature of their content rather than to protein concentration, as the observed differences in protein concentration between conditions did not show to be significant.

### 3.2. Applied Methods Allowed the Characterization of EVs by Their Protein Composition

The EVs can carry many different molecules, such as proteins [1,2]. In addition, previous studies have shown that fungal EVs carry different kinds of proteins, including those related to virulence [4]. To determine the potential function of EVs in the *B. cinerea* infection process, the protein content of EVs fraction and its controls (mycelium and supernatant control) obtained under GLU and TCW conditions were analysed by LC-MS. The mass spectrometry proteomics data have been deposited in the ProteomeXchange Consortium via the PRIDE partner repository [26], with the dataset identifier PXD040614. A total of 3442 proteins were identified after filtration (Supplementary Table S1).

**Figure 2:**
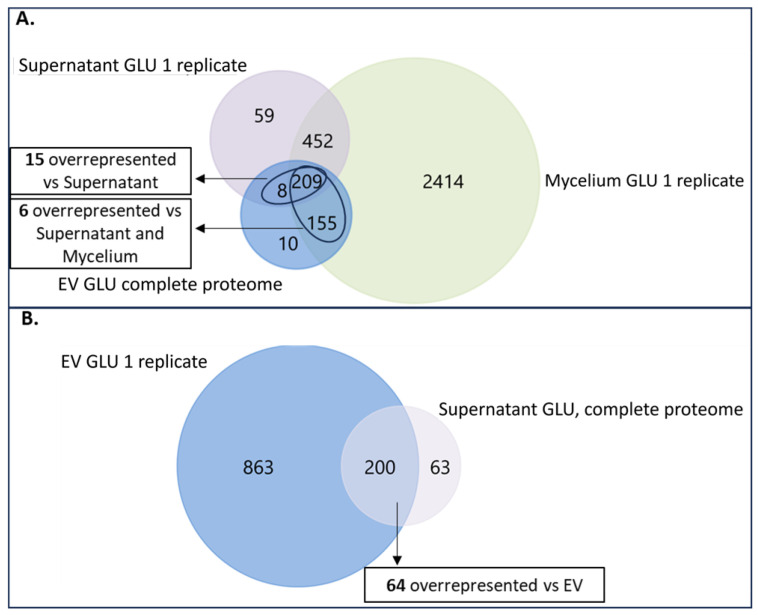
Venn diagrams of identified proteins under GLU condition. (**A**) Venn diagram of proteins presented in 3/3 replicates of EVs GLU fraction (EV GLU complete proteome), proteins presented in at least 1 replicate of Supernatant GLU fraction (Supernatant GLU 1 replicate) and proteins presented in at least 1 replicate of Mycelium GLU fraction (Mycelium GLU 1 replicate); (**B**) Venn diagram of proteins presented in 3/3 replicates of Supernatant GLU fraction (Supernatant GLU complete proteome), proteins presented in at least 1 replicate of EVs GLU fraction (EV GLU 1 replicate).

Following this, we performed the analysis of the proteins identified under each assayed condition, using the supernatant controls to eliminate possible protein contaminants from EVs proteome. Under GLU, 382 proteins were identified in 3/3 replicates of EVs fraction (Supplementary Table S2. From them, 165 proteins were not presented in any of the replicates of its supernatant control fraction, being exclusive for EVs under GLU (Figure 2A). In addition, 15 more proteins were found to be overrepresented with respect to the supernatant control (Figure 2A and Supplementary Figure S2), making a total of 180 exclusive and overrepresented proteins in EVs GLU versus Supernatant control GLU (exclusive and overrepresented EVs GLU proteome) (Supplementary Table S2). From these proteins, 16 proteins were also exclusive or overrepresented versus the mycelia fraction (Figure 2A and Supplementary Table S2). Aligned with a recent comprehensive study of potential marker proteins for *Candida albicans* and recommendations for marker proteins by the International Society of Extracellular Vesicles [42,43], these proteins could be good candidates for EVs markers under GLU conditions. In particular, 10 exclusive proteins were not detected in secreted nor in mycelia samples. 60% of these proteins were predicted to belong to MISEV2018 categories 1 or 2. 63 proteins were identified in all the replicates of Supernatant control GLU, and none of the replicates of its EVs fraction (Figure 2B), taken them as exclusive proteins of Supernatant control fraction under GLU. After the addition of overrepresented proteins (Figure 2B and Supplementary Figure S2), a total of 127 exclusive or overrepresented proteins were retained for further analysis (exclusive and overrepresented Supernatant GLU proteome) (Supplementary Table S2).

**Figure 3:**
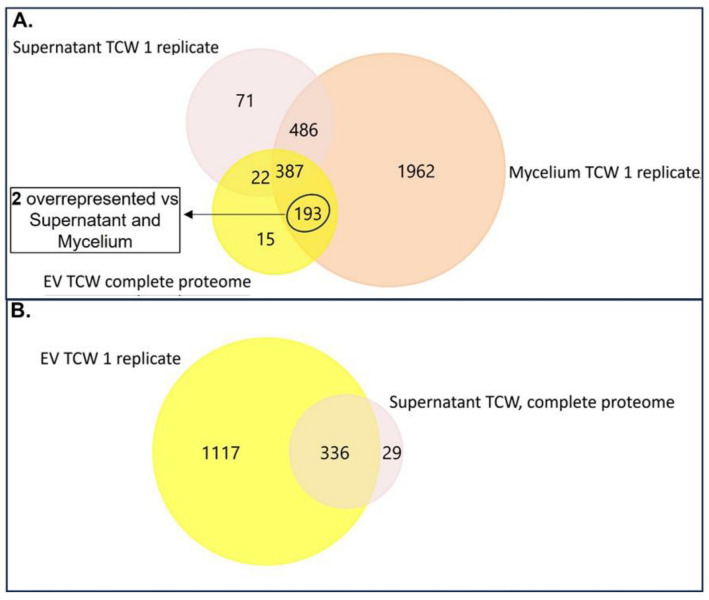
Venn diagrams of identified proteins under TCW condition. (**A**) Venn diagram of proteins presented in 3/3 replicates of EVs TCW fraction (EV TCW complete proteome), proteins presented in at least 1 replicate of Supernatant TCW fraction (Supernatant TCW 1 replicate) and proteins presented in at least 1 replicate of Mycelium TCW fraction (Mycelium TCW 1 replicate); (**B**) Venn diagram of proteins presented in 3/3 replicates of Supernatant TCW fraction (Supernatant TCW complete proteome) and proteins presented in at least 1 replicate of EVs TCW fraction (EV TCW 1 replicate). Note that there were not overrepresented proteins in EVs TCW compared to Supernatant TCW, so exclusive and overrepresented proteins in EVs TCW and Supernatant TCW were just exclusive proteins.

Under TCW, 617 proteins were identified in 3/3 replicates of EVs fraction (Supplementary Table S3). From them, 208 proteins were exclusive or were overrepresented compared to the supernatant control fraction under TCW (exclusive and overrepresented EVs TCW proteome) (Figure 3A, Supplementary Figure S2 and Table S3). Of these proteins, 17 out of 208 were also exclusive or over-expressed in relation to the mycelia fraction (Figure 3A and Supplementary Table S3). Following the recommendations for marker proteins previously mentioned [42,43], these 17 proteins could be good candidates for EVs markers under TCW conditions. In particular, 15 exclusive proteins were not detected in secreted nor in mycelia samples. Moreover, 53% of these proteins were predicted to belong to MISEV2018 categories 1 or 2. In addition, five proteins (A0A384J6L9, A0A384JM80, A0A384JSM9, A0A384JU06, A0A384JYC5) were identified as exclusive EVs proteins (not presented in the supernatant control or mycelia) in both conditions and could be good markers for EVs of *B. cinerea* in any other assayed condition. These proteins have been predicted to be unconventionally secreted effectors (A0A384J6L9 and A0A384JYC5), transmembrane proteins of the cell membrane or lysosome (A0A384JM80), and nuclear proteins with GPI membrane association (A0A384JSM9 and A0A384JU06). In addition, GO analysis of molecular function showed that A0A384JM80 presents ABC-type transporter activity and A0A384J6L9 oxidoreductase activity. Only one of these proteins has not been identified as uncharacterised protein, Bctaf5 (A0A384JU06), a transcription initiation factor TFIID subunit previously identified in other fungal EVs “http://exve.icc.fiocruz.br/proteins” (accessed on 20 August 2022). Finally, 29 proteins were identified as exclusive or overrepresented proteins of Supernatant control TCW (Supplementary Table S3).

The total number of proteins identified in our study of *Botrytis cinerea* EVs (382 and 617) was greater than the most common number of proteins identified in other fungal EVs studies, which reported fewer than 100 proteins. The latest best proteomics approaches performed with fungal EVs fall into two bands, between 100 and 500 and greater than 500, depending on the isolation method, the LC-MS technology used, and the applied statistics filter. So, our results of total protein identification are higher than those obtained in most fungi approaches [15] and similar to the recently reported one in *B. cinerea* (673 proteins) [7]. The number of high-confidence proteins selected as exclusive or overrepresented for EVs vs. the Supernatant control fraction (182 and 208) is in the range of protein numbers reported for the latest fungal studies that use supernatant as a control, such as *Zymoseptoria tritici* and *Fusarium oxysporum* [19,44]. To determine the specific role of these proteins in the infectious cycle, different molecular approaches will be developed in our laboratory for further analysis of selected proteins during functional prediction.

### 3.3. In Silico Analysis Corroborates the Isolation of High-Purity EVs Fraction

Following “The International Society for Extracellular Vesicles” (ISEV) guidelines, analysis of protein secretion and topology was performed using exclusive and overrepresented EVs proteomes to demonstrate the purity of EVs fraction [42]. Firstly, the presence of a lipid bilayer in the EVs fraction must be demonstrated by the identification of at least one transmembrane or GPI-anchored extracellular protein. This point has been predicted with the presence of a higher percentage of TMHMM proteins in the EVs fractions than in the supernatant control of both conditions (Table 1). Secondly, in all EVs of eukaryotes, the lipid bilayer encloses cytosolic material. So, to demonstrate that the EVs fraction contains more than open cell fragments, the subcellular localization of identified proteins was analysed using DeepLoc [37] (Table 1). The results showed a higher percentage of cytosolic proteins in the EVs fraction than in the supernatant control fraction. In addition, the identification of proteins from subcellular compartments other than the plasma membrane and endosomes has been previously identified in certain EVs subtypes of eukaryotes [42]. Other evidence to highlight is the increase in the percentage of the unconventionally secreted protein in both EVs fractions compared to the supernatant control. Among the mechanisms operative in unconventional secretion, EVs have been described as one of the unconventional secretion pathways of functionally relevant proteins [45]. In this work, we have identified 44 and 59 unconventionally secreted proteins in EVs GLU and EVs TCW exclusive and overrepresented proteomes, respectively (Supplementary Table S4).

Moreover, proteins with predicted functional activities may be detected in EVs. Many fungi secrete effector proteins to facilitate plant infection. “EffectorP” is a machine-learning method for fungal and oomycete effector prediction in secretomes [38]. The analysis performed with Effector P returned the identification of 89 proteins (42.8%) in EVs TCW exclusive/overrepresented proteome and 70 proteins (38.5%) in EVs GLU exclusive/overrepresented proteome. However, EffectorP analysis of previous *B. cinerea* EVs isolated from a solid medium with a cellophane layer could not identify any effector protein [7]. These results enhance our approach conditions as good inductors of the infective process and corroborate the adaptation of EVs cargo depending on the culture condition.

Finally, exclusive or overrepresented proteins identified in EVs GLU and EVs TCW were compared with (i) previously reported fungal EVs proteomes included in the web repository created for fungal EVs datasets, named ExVe “http://exve.icc.fiocruz.br” (accessed on 20 August 2022) [46]; and (ii) with the database vesiclepedia “http://microvesicles.org/index.html” (accessed on 20 August 2022), which contains molecular data identified in mammalian, fungi and bacteria [47]. Under glucose conditions, EVs exclusive and overrepresented proteome showed 92.3% proteins that were previously described in the ExVe or Vesiclepedia database (same gene/protein name or same predicted function/conserved domains). Of the 14 proteins that were not previously described in either of the databases, 6 were proteins belonging to the same biological process of proteins included in ExVE or Vesiclepedia. Under TCW condition, EVs proteome showed 87% proteins that were previously described in the ExVe or Vesiclepedia database. From the 27 proteins that were not previously described in either of the databases, 11 were proteins belonging to the same biological process of proteins included in ExVE or Vesiclepedia. These results point out a good selection of protein filtering for EVs proteome in both conditions, with the identification of proteins typically isolated in EVs of other organisms.

Finally, another proof of the purity of EVs in the selected fractions is the identification of some potential fungal EVs markers: (i) the 14-3-3 protein as exclusive protein in the EVs fraction of both conditions (not identified in the supernatant); and (ii) two HSP70 family member in the complete proteome of both conditions. Additionally, A0A384JCX1 (Heat shock 70 kDa protein/BCIN_03g06600) was identified as exclusive or overrepresented protein in EVs GLU, and A0A384JG51 (Bcsks2, an HSP70 family member) as exclusive or overrepresented protein in EVs TCW. 14-3-3 proteins have been proposed as EVs markers in the fungal phytopathogen *Colletotrichum higginsianum* [6]. At the same time, the Hsp70 Pfam domain is the unique orthologous group that has been identified in all fungal EV analyses [46]. Moreover, seven negative markers have been proposed for *Candida albicans*, from a list of exclusive and significantly enriched WCL (whole cell lysate): Lpd1, Sod2, Apr1, Cpy1, Lap41, Gpm1, and Abp1 [43]. We have found orthologous of most of these proteins underrepresented in EVs compared to mycelia under both condition: A0A384JA37 (Dihydrolipoyl dehydrogenase/Lpd1 orthologous), A0A384K4P4_(Bcabp1/Abp1 orthologous), A0A384JAC8 (the vacuolar aspartic proteinase Bcap2/Apr1 orthologous) [48], A0A384JUZ5 (The carboxypeptidase Bccp4/Cpy1, orthologous), A0A384J8G7 and A0A384JUH9 (the metalloaminopeptidases Bcape1 and Bcape4/Lap41 orthologous), and A0A384JFN5 (the Phosphoglycerate mutase BCIN_04g05860/Gpm1 orthologous) [43].

### 3.4. B. cinerea EVs Have a Functional Protein Profile Distinct from Supernatant Control, and It Is Also Adapted to the Environmental Condition

To better understand the potential function of the EVs in *B. cinerea*, a comparative proteomic study in each condition was performed using Gene Onthology (GO) annotations with the FunRich tool [28]. First, enrichment analysis of each fraction was calculated comparing EVs proteome against *B. cinerea* proteome (background). Secondly, fold enrichment analysis was performed comparing EVs fractions of each condition based on FunRich analysis software.

**Figure 4:**
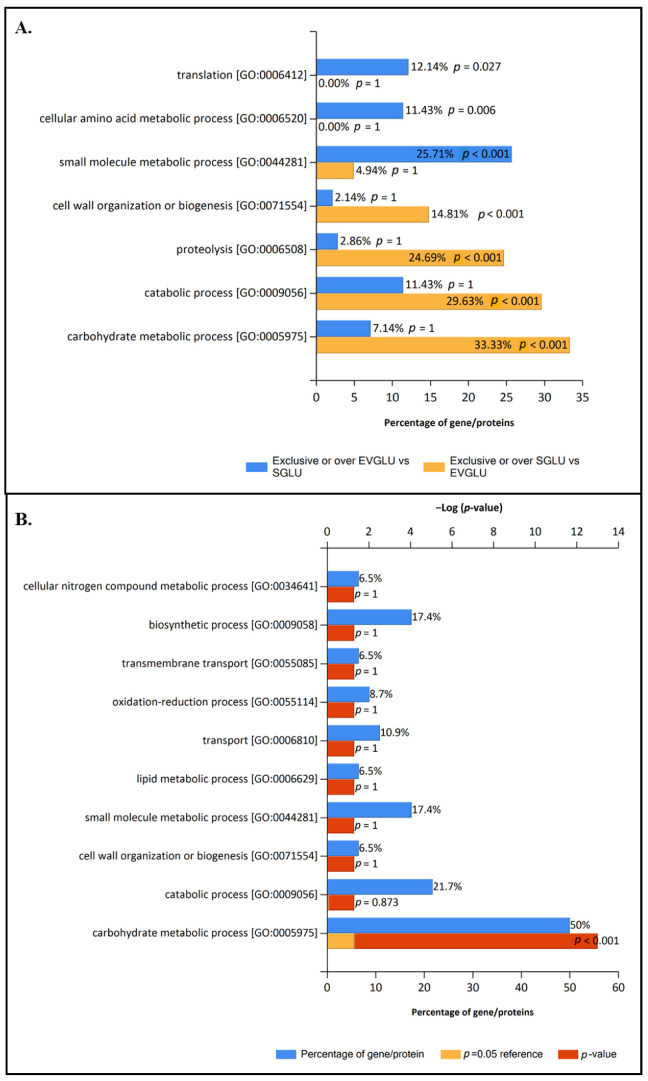
Enriched biological process GO in EVs GLU. Enrichment analyses were calculated using: (**A**) exclusive or overrepresented proteins presented in EVs GLU fraction (compared to supernatant control) (EVGLU) and in supernatant control GLU fraction (compared to EVs) (SGLU); and (**B**) common-nonregulated proteins presented in EVs GLU fraction and in supernatant control GLU fraction and comparing them with the background (*B. cinerea* proteome from Uniprot).

Enrichment analysis of exclusive and overrepresented proteins of EVs and Supernatant control fraction under GLU condition (Supplementary Tables S2 and S3) were performed to have a more concrete view of the specific functions of each fraction under that culture condition (Figure 4B). The results showed that translation, small molecule metabolic process, and amino acid metabolic process were enriched categories of EVs GLU exclusive or overrepresented proteome (Figure 4A).

In addition, to have more information about the possible role of EVs under GLU, enrichment analysis of common non-regulated proteins of EVs and Supernatant fraction under this condition were performed (Figure 4B). The analysis showed that the carbohydrate metabolic process was enriched in common non-regulated proteins (Figure 4B) as well as in the supernatant (Figure 4), but it was not enriched in exclusive and overrepresented proteins of EVs GLU (Figure 4A). This means that carbohydrate metabolism is a biological function shared between EVs and Supernatant under GLU conditions but with higher involvement of the last one.

**Figure 5:**
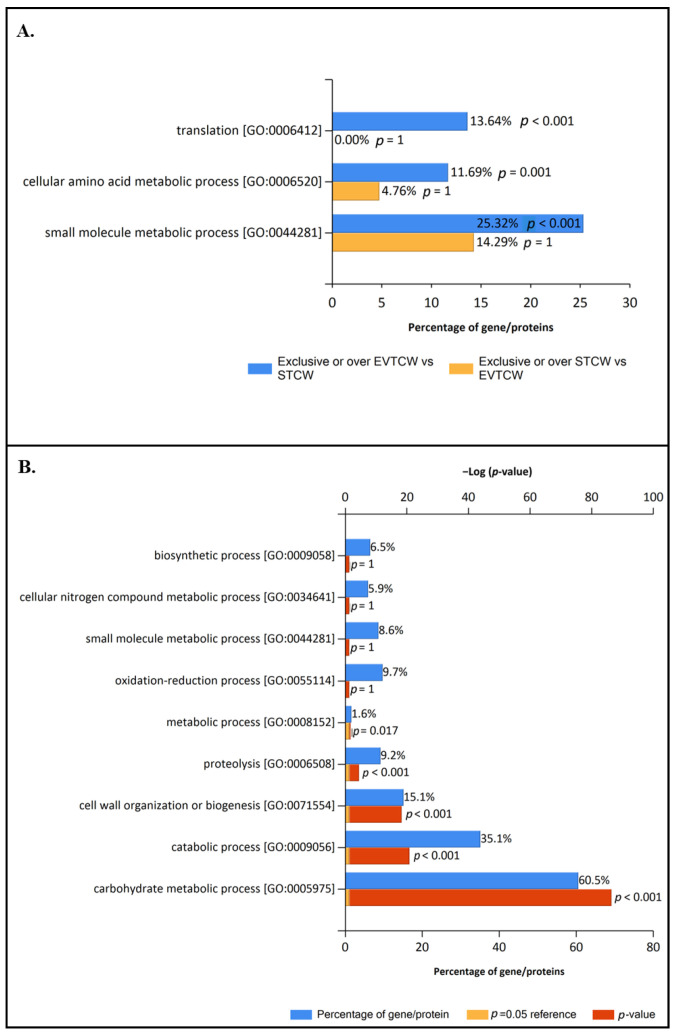
Enriched biological process GO in EVs TCW. Enrichment analyses were calculated using: (**A**) exclusive or overrepresented proteins presented in EVs TCW fraction (compared to supernatant control) (EVTCW) and in supernatant control TCW fraction (compared to EVs) (STCW); and (**B**) common-non regulated proteins presented in EVs TCW fraction and in supernatant control TCW fraction and comparing them with the background (*B. cinerea* proteome from Uniprot).

The same GO analyses were carried out in fractions under TCW conditions (Figure 5). Enrichment analysis using exclusive and overrepresented proteins of EVs and Supernatant fractions was performed (Figure 5A). This analysis showed EVs TCW enrichment of translation, small molecule metabolic process, and cellular amino acids metabolic process, just like in EVs GLU (Figure 5A). This result highlights the crucial role of EVs in these three biological functions. Moreover, our results agree with previously reported GO analysis in plant–pathogenic fungal EVs proteomes, such as *F. oxysporum*, *Z. tritici* [19,20], and *B. cinerea* growing on cellophane [7].

Unlike the GLU condition, when common non-regulated proteins of EVs TCW and Supernatant TCW were analysed (Figure 5B), carbohydrate metabolism, proteolysis, and cell wall metabolism were presented as enriched BP GO categories. However, these categories were not enriched in exclusive or over-expressed proteins in EVs TCW, nor were they in supernatant TCW fraction (Figure 5A). That analysis pointed out that carbohydrate metabolism, proteolysis, and cell wall degrading activity under TCW conditions was shared by EVs and supernatant fractions. These categories were also identified in EVs isolated from *B. cinerea* growing on a solid medium with a cellophane layer, but in that study, no supernatant control was performed to filter out the potential contamination of conventionally secreted proteins [7]. So, whether the presence of these activities in both fractions is due to contamination from disrupted EVs or due to the secretion of proteins via EVs and classical secretion pathways will require further investigation. On this matter, it is interesting to highlight that some kinds of protein can be secreted by conventional and unconventional secretory pathways under certain conditions [49]. In addition, accumulated evidence points to EVs as responsible for the unconventional secretion of cell wall-modifying enzymes in fungi [50]. Moreover, these BP GO categories have been previously reported to be implicated in the *B. cinerea* infection process, such as the infection cushion production [51]. Joining this evidence and our GO results, we can conclude that some essential proteins in the infection process of *B. cinerea* are very likely secreted by the fungus using two pathways of secretion under TCW condition, the conventional (secretome) and the unconventional (EVs), reinforcing the infection machinery.

**Figure 6:**
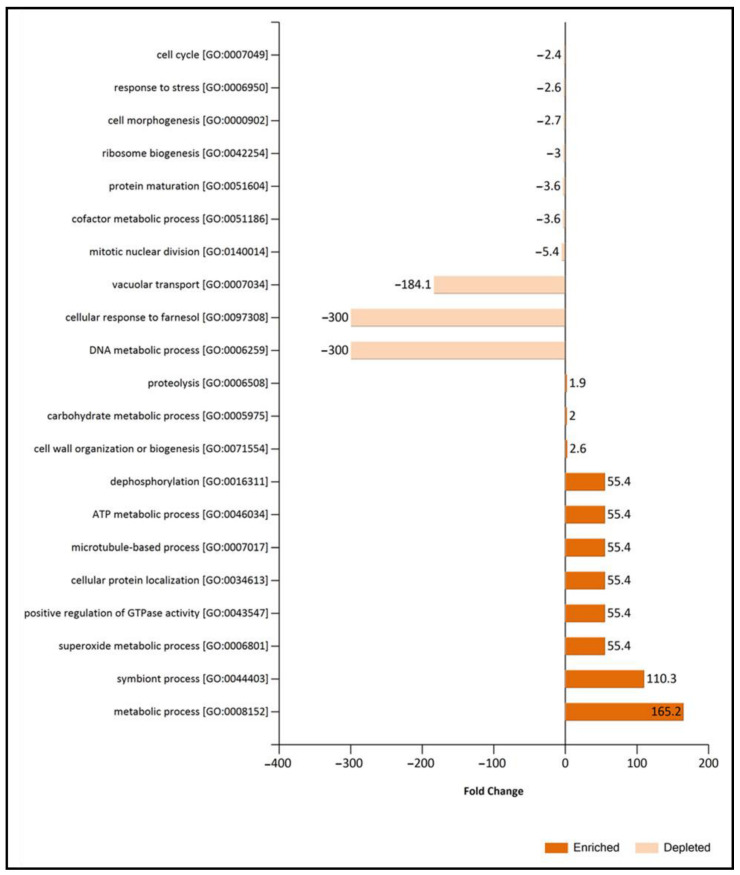
Fold change analysis of Biological Process GO categories in relevant EVs TCW and EVs GLU proteomes. Fold Change were calculated using Exclusive or over-expressed proteins in EVs plus common-nonregulated proteins of EVs and Supernatant control in TCW (relevant EVs TCW proteome) and comparing them with relevant EVs GLU proteome. Depleted proteins are those enriched in relevant EVs TCW proteome.

**Figure 7:**
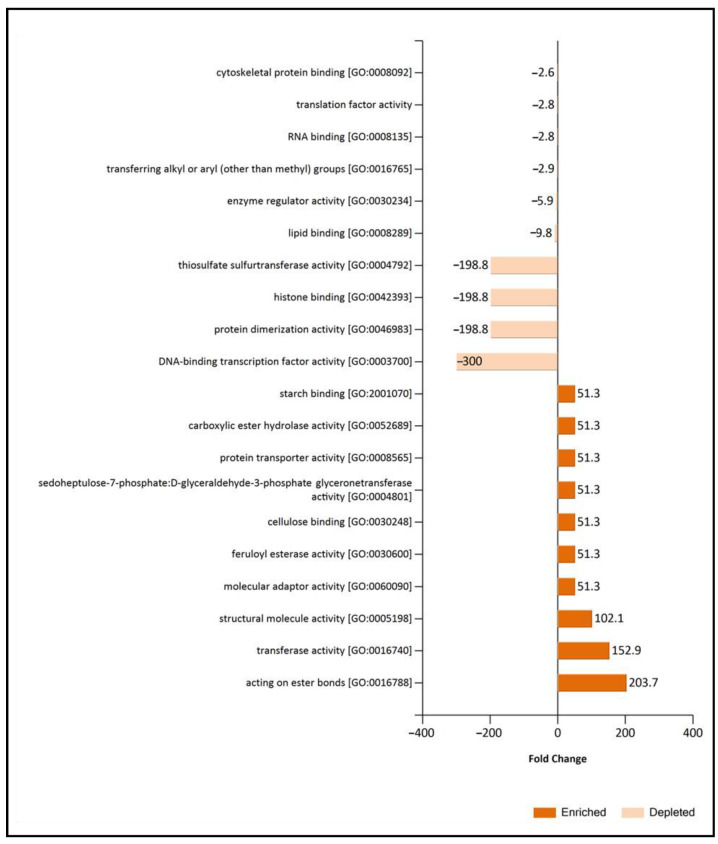
Fold change analysis of Molecular Function GO categories in relevant EVs TCW and EVs GLU proteomes. Fold Change were calculated using Exclusive or over-expressed proteins in EVs plus common-nonregulated proteins of EVs and Supernatant control in TCW (relevant EVs TCW proteome) and comparing them with relevant EVs GLU proteome. Depleted proteins are those enriched in relevant EVs GLU proteome.

Considering that common non-regulated proteins of EVs and supernatant control have shown an essential role in the description of the EVs, they were merged with exclusive and overrepresented proteins of EVs fraction (relevant EV proteome) to further compare the function of the EVs between conditions using Fold enrichment analysis of Biological process GO (Figure 6) and molecular function GO categories (Figure 7). Fold enrichment results corroborated the increase of cell wall metabolism and proteolysis under TCW conditions, while categories related to transcription, translation, and lipid metabolism were enriched in EVs GLU. Focusing on BP GO categories, the symbiont process and superoxide metabolic process were also enriched under TCW compared to the GLU condition. However, cellular response to farnesol was BP GO categories enriched under GLU condition (Figure 6).

Symbiont process and superoxide metabolic process categories identified as enriched in EVs TCW proteome were represented by: (i) Bcspl1 (A0A384JBC5) and the Heat shock protein SSB1 (A0A384JG51) in Symbiont process; and (ii) Superoxide dismutase 1 copper chaperone Bcccs1 (A0A384JDH6) in superoxide metabolic process. The cerato-platanin BcSpl1 has been reported to contribute to *B. cinerea* virulence by association with the plant plasma membrane causing disorganization of chloroplasts and necrosis. BcSpl1 was found as an abundant protein in the secretome of *B cinerea* induced with plant extracts and even more abundant in the fungal cell wall of this fungi [52,53]. Recently, it has been shown that EVs from *S. cerevisiae* contain cell wall-related proteins [54]. Our approach shows that BcSpl1 isolation in *B cinerea* secretome may be due to a co-isolation of secreted and EVs protein during extraction protocols. Moreover, these data suggest that BcSpl1 production must involve and/or interact with EVs. So, it is reasonable that BcSpl1 is presented in the cell wall and EVs of *B. cinerea*, allowing its transport to the plant plasma membrane to perform its necrotizing action. SSB1 has been previously reported as a chaperone that plays a dual role in de novo protein folding and ribosome biogenesis in the yeast *S. cerevisiae* [55]. The ortholog of SSB1 in *B. cinerea* is Bcsks2, an HSP70 family member [56]. Interestingly, the Hsp70 domain was the only ortholog group found in the EVs proteome of all fungal species deposited in ExVe. Moreover, this protein was also found in EVs GLU (Supplementary Table S2) as a common-non-regulated protein of EVs in both conditions suggesting that this family of heat-shock proteins might play a universal role in fungal EVs [46]. Heat shock proteins seem to be essential for the formation of fungal EVs and also for stress response and survival under adverse environmental conditions [46]. So, Bcsks2 could be implicated in *B. cinerea* Evs’ biogenesis or stress response during the infection process. In addition, a homolog of SSB1 in *Magnaporthe oryzae* (MoSsb1) mediated Cell-Wall Integrity Signalling, growth, and pathogenicity [56], pointing out Bcsks2 as a potential pathogenic factor in *B. cinerea*. Here we have found these proteins as common non-regulated proteins of EVs and Supernatant control (Supplementary Table S3). Finally, The Superoxide dismutase 1 copper chaperone Bcccs1 may be required for the activation of Sod1 in response to the toxic effects of reactive oxygen species (ROS) generated by host defense during the infection process, just as other fungi [57]. Moreover, the action of Sod1 is required for virulence in *Candida albicans* [58], highlighting Bccs1 as a potential virulence factor in *B. cinerea*.

On the contrary, cellular response to farnesol was one of the most enriched BP GO categories under GLU conditions in EVs. Farnesol is an isoprenoid intermediate in the mevalonate (MVA) pathway that has been reported to induce a toxic effect in *B. cinerea* [59]. One of the proteins categorized within this BP GO is a putative CDP-alcohol phosphatidyltransferase, BcPio5 [60]. Orthologs of these proteins have been reported to participate in phospholipid homeostasis, which plays an important role in fungal development, fungicide resistance, and virulence in *Fusarium graminearum* [61]. This evidence highlights the potential role of EVs in the transport of virulence factors during the infective process.

All these results are consistent with previous analysis of the group, where it was reported that Under GLU condition, the mevalonate pathway is upregulated due to the production of toxins (botryoidal and dihydrobotrydial) [12]; and under TCW condition, the secretion of cell wall degrading enzymes was upregulated [12]. The difference in BP enrichment of EVs in both assayed conditions reveals a connection between the cargo and the environmental signals, which is essential to host adaptation during the infection process. Moreover, other authors have previously reported EVs proteome changes depending on the growth medium *Fusarium oxysporum* f. sp. *vasinfectum* [44] and *Histoplasma capsulatum* [41], corroborating our results.

### 3.5. KEGG Analysis Reinforce the Involvement of B. cinerea EVs in the Infection Process

**Figure 8:**
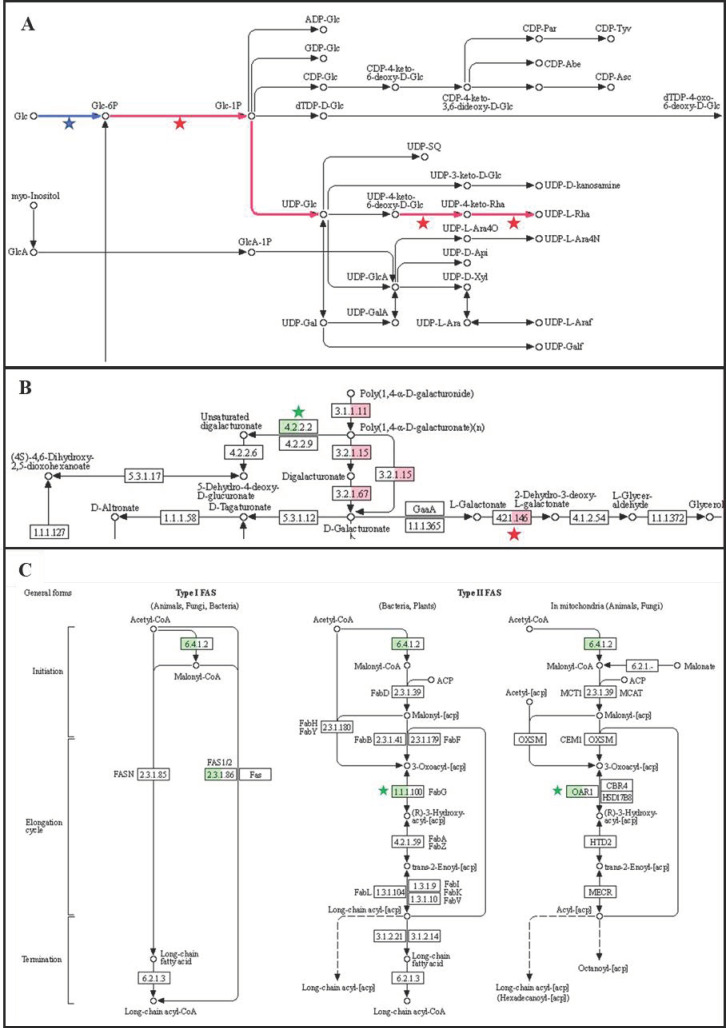
Most representative KEGG pathways of EVs TCW and EVs GLU. KO annotation of EVs TCW and EVs GLU relevant proteomes were used in Reconstruct KEGG mapper tool. (**A**) Detailed of UDP-glucose and UDP-L-Rha biosynthesis from map01250 “Nucleotide sugar biosynthesis”; (**B**) Detailed of pectin degradation from map00040 “Pentose and Glucuronate Interconversion”. (**C**) Detailed from map00061 “Fatty acid biosynthesis”. Blue: under TCW and GLU; Red: Under TCW; Green: under GLU; star: identified as exclusive or overrepresented protein in EVs proteome versus supernatant control of this condition. Pectin: Poly(1,4-alpha-D-galacturonide). Pectate: Poly(1,4-alpha-D-galacturonate).

In order to highlight all the differences in the function of EVs isolated from GLU and TCW conditions, a KEGG analysis of EV fractions in both conditions was performed. KEGG reconstruct pathway was performed with the K number assigned by BlastKOALA and KoFAMKOala for relevant and exclusive/overrepresented EVs proteomes under both conditions (Supplementary Table S5). This analysis returned massive information about potential pathways where EVs could be implicated (Supplementary Table S5), and most of them validate our GO analysis. We have focused on those pathway modules (functional units of gene sets in metabolic pathways, including molecular complexes) that are almost completely covered by the identified proteins or those marking differences between conditions. Under TCW conditions, EVs relevant proteome presented as the most relevant pathway pectin degradation and nucleotide sugar biosynthesis (UDP-Glucose, and UDP-L-Rhamnose) (Figure 8A,B). On the other hand, fatty acid biosynthesis, initiation, and elongation were identified under GLU conditions. (Figure 8C).

It has been previously reported that UDP-sugars in fungi are incorporated into secondary metabolites and are used for the detoxification of plant defense molecules and to produce fungal cell wall glycans [62]. Moreover, the alteration of biosynthesis of UDP-L-Rhamnose adversely affects virulence, colonization, and pathogenicity in plant–pathogenic fungi [63]. In this study, we have identified three proteins implicated in this pathway (A0A384JXF3, A0A384J564, A0A384JJU9) (Figure 8A and Supplementary Table S5). In *Botrytis cinerea*, two genes have been involved in the production of UDP-L-Rhamnose from UDP-Glucose: (i) the gene *bcdh*, a UDP-glucose-4,6-dehydratase (F8U971); and (ii) the gene *bcer*, UDP-4-keto-6-deoxyglucose-3, 5-epimerase/-4-reductase (F8U972). The *Δbcer* strain showed reduced virulence of *B. cinerea* [62]. In this work, we have found as exclusive protein in EVs TCW Bcer (A0A384JJU9) (Figure 8A), highlighting again the role of vesicles in the transport of virulence factors and, therefore, their implication in the infective process of the fungus. Moreover, it is well reported that some fungal EVs functions are cell wall biosynthesis and stress response [4]. So, Bcer could be involved in the cell wall biosynthesis or remodeling and detoxification of plant molecules in the zone of infection through its transport by EVs. In addition, it is known that effective pectin degradation is important for the virulence of *B. cinerea* [64,65]. Most of the reported *B. cinerea* pectin degrading enzyme activities have been detected under TCW conditions in the common non-regulated protein of EVs fraction and supernatant control (Figure 8B and Supplementary Tables S3 and S5) [66]: 1. The pectin esterase Bcpme1 [EC:3.1.1.11] (A0A384JQ57); 2. The pectin esterase domain-containing protein [EC:3.1.1.11] (A0A384K263); 3. The endo-polygalacturonase (endo-PG) Bcpg6 [EC3.2.1.15] (A0A384JAG7); 4. The endo-PG Bcpg4 [EC3.2.1.15] (A0A384JBT3); 5. The endo-PG Bcpg3 [EC3.2.1.15] (A0A384JFT4); 6. The endo-PG Bcpg1 [EC3.2.1.15] (A0A384K208); and 7. 5 galacturan 1,4-alpha-galacturonidase [EC:3.2.1.67], including Bcpgx1 (A0A384J688, A0A384J801, A0A384JHC7, A0A384JJG2, A0A384JVT2, A0A384JZU9). The monosaccharide d-galacturonic acid is the major component of pectin and, consequently, is the final product released from pectin degradation. The d-galacturonic acid catabolic pathway in *B. cinerea* consists of three catalytic steps involving two non-homologous galacturonate reductase genes (*bcgar1* and *bcgar2*), a galactonate dehydratase gene (*bclgd1*) and a 2-keto-3-deoxy-l-galactonate aldolase gene (*bclga1*) [66]. Knockout mutants in each of the three catalytic steps were affected by virulence [67]. Bclgd1 (A0A384J6U1/EC:4.2.1.146) has been found as an exclusive protein in EVs TCW not identified in the supernatant control nor in EVs GLU. For the complete catabolism of d-galacturonic acid, three enzymes were missing, Bcgar1, Bcgar2, and Bclga1. However, Bcgar1 (A0A384JKV9) was identified in 2/3 of EVs TCW fraction and in none of EVs GLU or supernatants control fractions (Supplementary Table S3), being considered an EVs TCW exclusive protein too. So, what can be clearly stated is that EVs under TCW conditions transport essential proteins in the catabolism of pectin, pointing to their potential role in the virulence of the fungus in combination with the supernatant.

Interestingly, from these two essential pathways (UDP-L-Rhamnose biosynthesis and pectin degradation), only Bcdh (not identified in our study) and the pectin esterase domain-containing protein (A0A384K263) have been previously identified in *B. cinerea* EVs isolated from cellophane containing medium [7], which highlights the adaptation of EVs cargo to the environmental condition.

On the contrary, the fatty acid de novo biosynthesis pathway was identified in EVs GLU (Figure 8C). The synthesis of fatty acids (FAs) plays important roles during the infection process in plant pathogenic fungi, such as the alteration of fatty acid composition [68]. FAs are the first step for the generation of complex lipids, which have been reported as essential molecules in the production of toxins [68]. In addition, we have identified in EVs GLU the last enzymatic step involved in the biosynthesis of isopentenyl pyrophosphate (IPP) via the mevalonate (MVA) pathway, the enzyme kinase (Bcmvd1/A0A384JEF0) (Supplementary Tables S2 and S5). IPP is the first precursor in the biosynthesis of terpenes in fungi, including toxins [69]. This result is consistent with our previous knowledge of glucose as an inductor of toxins production in *B. cinerea* [12]. Furthermore, this could indicate that EVs transport the precursors of *Botrytis* toxins to the plant to reinforce their production in the zone of infection.

Next, some interesting common KEGG pathways in both conditions, but not represented by the same identified proteins, were: (i) one carbon pool by folate (Supplementary Figure S3); (ii) Biosynthesis of cofactors (Supplementary Figure S4A); (iii) antioxidant system (Supplementary Figure S4A); and (iv) signalling (Supplementary Table S5).

All these pathways have been previously reported to be implicated in virulence or pathogenesis [70,71,72]. In the folate pathway, we have identified under both conditions different enzymes for the conversion of 5,6,7,8-Tetrahydrofolate (THF) to 5,10-Methylenetetrahydrofolate (5,10-Methylene-THF) (Supplementary Figure S3). The reduction of 5,10-Methylene-THF to 5-methyltetrahydrofolate is required for methionine biosynthesis, which has been reported as an essential reaction for the pathogeny of some plant–pathogenic fungi [70]. Secondly, Under TCW conditions, we identified two enzymes for biotin production. It is known that fungal biotin homeostasis is essential for host immune evasion and virulence during infection [71]. Thirdly, effective antioxidant systems are necessary to balance the intracellular redox state, including the thioredoxin and the glutathione system. Furthermore, catalases, superoxide dismutase, and peroxidases eliminate ROS by enzymatic inactivation [72]. The interplay of all these enzymes is necessary to obtain a stable redox environment that allows a successful infection process [72]. In *B. cinerea* EVs, we have identified different antioxidants enzymes under each condition (Supplementary Table S5): (i) the superoxide dismutase BcSod4 (A0A384JHR7), the Thiol-specific peroxidase Bcprx9 (A0A384JUA9) and the glutathione synthase Bcgsh2 (A0A384JTX1) in EVs TCW; and (ii) the catalase Bccat5 (A0A384JBF3) and the glutathione reductase (NADPH) Bcglr2 in EVs GLU. Most of these enzymes have been previously reported to be upregulated under ROS stress induction by antifungal compounds [72,73]. Of the above-mentioned proteins, only Bcglr2 has been previously tested, showing unaffected pathogenicity [74]. But other related proteins have been shown to affect virulence, such as Bcsod1 [75]. In summary, these results showed an activation of the antioxidant systems under both conditions, highlighting the relevant role of EVs in ROS stress defense during the different states of the infection process, early stage (TCW condition, during penetration) and later stage of infection (GLU, after penetration) [12]. In addition, new potential virulence/pathogenicity factors can be found between all the proteins mentioned above.

Finally, some differences in signaling proteins were observed. One of the most significant ones was the identification of GTP-Binding Proteins, which was also an overrepresented GO category in the EVs proteome of *Fusarium graminearum* [5]. In EVs TCW we found five Small (monomeric) G-proteins: A0A384JZS6 (Bcras1), A0A384JEP9 (Bcrho1), A0A384JKG7 (Bcrho3), A0A384J5T1 (BCIN_01g06220/Ran family GTP-binding nuclear protein), and A0A384JX46 (BCIN_11g04620/ADP-ribosylation facto). In addition, one Heterotrimeric G-protein was identified in EVs TCW and EVs GLU, A0A384JUU4 (Bcgg1), as a common protein in both kinds of EVs. This highlights a higher level of activation of Monomeric G proteins signaling pathway in EVs TCW. This family of signaling proteins plays pivotal roles in fungal virulence [76]. In Botrytis, Bcrho1 was not described as a virulence/pathogenicity factor yet. However, Rho1 was described as a virulence factor in *Aspergillus fumigatus* [77]. Moreover, it has been reported that BcRho3 was implicated in the regulation of mycelial growth, conidiation production, and virulence [78]. The ∆bcrho3 mutant has shown a reduction in virulence and impaired penetration ability in *B. cinerea* [78]. BCIN_11g04620 is an ADP-ribosylation factor family protein. This kind of protein has been reported to regulate the assembly of coat proteins on the Golgi and/or endosomes [79] and also as a critical regulator of the virulence in *F. oxysporum* [80]. Moreover, this protein has also been identified in EVs of *B. cinerea* isolated from solid medium containing cellophane [7]. All these results highlight BCIN_11g04620 as a new potential virulence factor in *B. cinerea,* pointing out again the relevance of EVs in the regulation of the infection process.

## 4. Conclusions

The current study represents the first comparative proteomic analysis of *B. cinerea* EVs generated under different pathogenic states. The results have proven that *B.cinerea* releases EVs showing differences in their morphology under both assayed conditions. That evidence was reflected in functional differences in GO and KEGG analysis, implying the EVs cargo adaptation to the environmental signals, which is essential to plant–pathogen interaction. In addition, GO analysis showed enrichment in cell wall metabolism and proteolysis in EVs under TCW, which was shared with secreted proteins. This result reveals close cooperation between conventional (secreted proteins) and unconventional (EVs) secretion pathways in crucial steps of the infective process. Moreover, KEGG analysis also corroborates the potential role of EVs during plant invasion, with the identification of many virulence factors and proteins implicated in crucial steps such as pectin degradation, nucleotide sugar biosynthesis, redox state, biotin production, cofactor metabolism, and signalling. Finally, between the identified proteins, we have highlighted new potential virulence/pathogenic factors, such as BcSks2, BcCcs1, or BcPio5. These proteins will be further analysed by different molecular approaches, including plant infection experiments.

## Figures and Tables

**Table 1:** Topology, secretion, and subcellular localization prediction analysis.

	TMHMM	SP	UPS	Intracellular	Localization
EVs GLU exclusive and overrepresented proteins	12.63%	6.59%	24.7%	56.08%	59.9% cytoplasm (53.21% lipidated)
					13.2% mitochondrion
					7.7% nucleus
					6% endoplasmic reticulum
					4.4% lysosome
					4.4% extracellular (50% lipidated)
					2.2% cell membrane
					1.64% peroxisome
					0.54% golgi
EVs TCW exclusive and overrepresented proteins	15.38%	2.4%	28.4%	53.85%	63% cytoplasm (52.7% lipidated)
					12% mitochondrion
					7.21% cell membrane
					6.7% nucleus
					4.8% endoplasmic reticulum
					2.9% lysosome/vacuole
					1.9% extracellular (50% lipidated)
					0.96% peroxisome
					0.5% golgi
Supernatant GLU exclusive and overrepresented proteins overrepresented proteins	3.9%	60.6%	6.3%	29.1%	74.8% extracellular (58.9% lipidated)
					16.5% cytoplasm (66.7% lipidated)
					3.1% lysosome/vacuole
					3.1% nucleus
					1.6% endoplasmic reticulum
					0.8% mitochondrion
Supernatant TCW exclusive and overrepresented proteins	6.89%	27.58%	24,13%	41.38%	51.7% cytoplasm (53.3% lipidated)
					20.7% extracellular (50% lipidated)
					10.3% endoplasmic reticulum
					6.9% mitochondrion
					6.9% cell membrane
					3.4% nucleus

TMHMM: prediction of transmembrane helices of integral membrane proteins. SP: presence of signal peptides prediction. UPS: Unconventionally secreted proteins prediction.

## Data Availability

The mass spectrometry proteomics data have been deposited to the ProteomeXchange Consortium via the PRIDE partner repository with the dataset identifier PXD040614.

## Supplementary Materials

The following supporting information can be downloaded at: https://www.mdpi.com/article/10.3390/jof9090872/s1. Figure S1: *B. cinerea* EVs at lower magnification (200 nm–500 nm); Table S1: Proteins identified after filtration of contaminants, only identified by site modification and reverse, and presented in at least two replicates of one group (replicates of 1 condition/fraction) with more than one peptide; Figure S2: Analysis on total protein distribution; Table S2: Proteins identified in EV fraction under GLU condition, Table S2A. Proteins identified in at least 3 replicates of EV fraction under GLU condition, Table S2B. Proteins identified as exclusive and overexpressed in EVs fraction versus Supernatant under GLU condition (exclusive and overexpressed Evs GLU proteome), Table S2C. Proteins identified as exclusive and overexpressed in EVs fraction versus Supernatant and Mycelium under GLU condition, Table S2D. Proteins identified as exclusive and overexpressed in Supernatant fraction versus EVs under GLU condition, Table S2E. Proteins identified as common-non regulated in EV and Supernatant fraction under GLU condition; Figure S3: Common KEGG pathway of EVs GLU and EVs TCW; Table S3: Proteins identified in EV fraction under TCW condition, Table S3A. Proteins identified in at least 3 replicates of EV fraction under TCW condition, Table S3B. Proteins identified as exclusive and overexpressed in EVs fraction versus Supernatant under TCW condition (exclusive and overexpressed EVs TCW proteome), Table S3C. Proteins identified as exclusive and overexpressed in EVs fraction versus Supernatant and Mycelium under TCW condition, Table S3D. Proteins identified as exclusive and overexpressed in Supernatant fraction versus EVs under TCW condition, Table S3E. Proteins identified as common-non regulated in EV and Supernatant fraction under TCW condition; Figure S4: Common KEGG pathway of EVs GLU and EVs TCW “Biosynthesis of cofactors” and “antioxidant system”; Table S4: Proteins identified as unconventionally secreted in exclusive and overexpressed EVs proteome, Table S4A: Proteins identified as unconventionally secreted in exclusive and overexpressed EVs TCW proteome, Table S4B: Proteins identified as unconventionally secreted in exclusive and overexpressed EVs GLU proteome; Table S5: K number assigned by BlastKOALA and KoFAMKOala of relevant and exclusive/overexpressed EVs proteomes, Table S5A: K number assigned by BlastKOALA and KoFAMKOala of relevant and exclusive/overexpressed EVs proteomes under GLU condition, Table S5B: K number assigned by BlastKOALA and KoFAMKOala of relevant and exclusive/overexpressed EVs proteomes under TCW condition.

## References

[1] Rybak K., Robatzek S. (2019). Functions of extracellular vesicles in immunity and Virulence. Plant Physiol..

[2] Rizzo J., Taheraly A., Janbon G. (2021). Structure, composition and biological properties of fungal extracellular vesicles. microLife.

[3] Takeo K., Uesaka I., Uehira K., Nishiura M. (1973). Fine Structure of *Cryptococcus neoformans* Grown in vitro as Observed by Freeze-Etching. J. Bacteriol..

[4] Liebana-Jordan M., Brotons B., Falcon-Perez J.M., Gonzalez E. (2021). Extracellular vesicles in the fungik. Int. J. Mol. Sci..

[5] Garcia-Ceron D., Lowe R.G.T., McKenna J.A., Brain L.M., Dawson C.S., Clark B., Berkowitz O., Faou P., Whelan J., Bleackley M.R. (2021). Extracellular Vesicles from *Fusarium graminearum* Contain Protein Effectors Expressed during Infection of Corn. J. Fungi.

[6] Rutter B.D., Chu T.T., Dallery J.F., Zajt K.K., O’Connell R.J., Innes R.W. (2022). The development of extracellular vesicle markers for the fungal phytopathogen *Colletotrichum higginsianum*. J. Extracell. Vesicles.

[7] De Vallee A., Dupuy J.W., Moriscot C., Gallet B., Vanderperre S., Guignard G., Rascle C., Calvar G., Malbert B., Gillet F.X. (2023). Extracellular Vesicles of the Plant Pathogen *Botrytis cinerea*. J. Fungi.

[8] Fillinger S., Elad Y. (2016). Botrytis—The Fungus, the Pathogen and Its Management in Agricultural Systems.

[9] Staats M., van Kan J.A. (2012). Genome update of *Botrytis cinerea* strains B05.10 and T4. Eukaryot. Cell.

[10] Atwell S., Corwin J.A., Soltis N.E., Subedy A., Denby K.J., Kliebenstein D.J. (2015). Whole genome resequencing of *Botrytis cinerea* isolates identifies high levels of standing diversity. Front. Microbiol..

[11] Urban M., Cuzick A., Rutherford K., Irvine A., Pedro H., Pant R., Sadanadan V., Khamari L., Billal S., Mohanty S. (2017). PHI-base: A new interface and further additions for the multi-species pathogen-host interactions database. Nucleic Acids Res..

[12] Escobar-Niño A., Morano Bermejo I.M., Carrasco Reinado R., Fernandez-Acero F.J. (2021). Deciphering the Dynamics of Signaling Cascades and Virulence Factors of *B. cinerea* during Tomato Cell Wall Degradation. Microorganisms.

[13] Lineiro E., Macias-Sanchez A.J., Espinazo M., Cantoral J.M., Moraga J., Collado I.G., Fernandez-Acero F.J. (2018). Phenotypic Effects and Inhibition of Botrydial Biosynthesis Induced by Different Plant-Based Elicitors in *Botrytis cinerea*. Curr. Microbiol..

[14] Rizzo J., Rodrigues M.L., Janbon G. (2020). Extracellular Vesicles in Fungi: Past, Present, and Future Perspectives. Front. Cell Infect. Microbiol..

[15] Bleackley M.R., Dawson C.S., Anderson M.A. (2019). Fungal Extracellular Vesicles with a Focus on Proteomic Analysis. Proteomics.

[16] Fernandez-Acero F.J., Colby T., Harzen A., Carbu M., Wieneke U., Cantoral J.M., Schmidt J. (2010). 2-DE proteomic approach to the *Botrytis cinerea* secretome induced with different carbon sources and plant-based elicitors. Proteomics.

[17] Vallejo I., Carbú M., Muñoz F., Rebordinos L., Cantoral J.M. (2002). Inheritance of chromosome-length polymorphisms in the phytopathogenic ascomycete *Botryotinia fuckeliana* (anam. *Botrytis cinerea*). Mycol. Res..

[18] English P.D., Jurale J.B., Albersheim P. (1971). Host-Pathogen Interactions: II. Parameters Affecting Polysaccharide-degrading Enzyme Secretion by *Colletotrichum lindemuthianum* Grown in Culture. Plant Physiol..

[19] Hill E.H., Solomon P.S. (2020). Extracellular vesicles from the apoplastic fungal wheat pathogen *Zymoseptoria tritici*. Fungal Biol. Biotechnol..

[20] Bleackley M.R., Samuel M., Garcia-Ceron D., McKenna J.A., Lowe R.G.T., Pathan M., Zhao K., Ang C.S., Mathivanan S., Anderson M.A. (2019). Extracellular Vesicles from the Cotton Pathogen *Fusarium oxysporum* f. sp. vasinfectum Induce a Phytotoxic Response in Plants. Front. Plant Sci..

[21] Gupta S., Rawat S., Arora V., Kottarath S.K., Dinda A.K., Vaishnav P.K., Nayak B., Mohanty S. (2018). An improvised one-step sucrose cushion ultracentrifugation method for exosome isolation from culture supernatants of mesenchymal stem cells. Stem. Cell Res. Ther..

[22] Rappsilber J., Ishihama Y., Mann M. (2003). Stop and Go Extraction Tips for Matrix-Assisted Laser Desorption/Ionization, Nanoelectrospray, and LC/MS Sample Pretreatment in Proteomics. Anal. Chem..

[23] Cox J., Mann M. (2008). MaxQuant enables high peptide identification rates, individualized p.p.b.-range mass accuracies and proteome-wide protein quantification. Nat. Biotechnol..

[24] Tyanova S., Temu T., Cox J. (2016). The MaxQuant computational platform for mass spectrometry-based shotgun proteomics. Nat. Protoc..

[25] Tyanova S., Temu T., Sinitcyn P., Carlson A., Hein M.Y., Geiger T., Mann M., Cox J. (2016). The Perseus computational platform for comprehensive analysis of (prote)omics data. Nat. Methods.

[26] Perez-Riverol Y., Bai J., Bandla C., García-Seisdedos D., Hewapathirana S., Kamatchinathan S., Kundu D.J., Prakash A., Frericks-Zipper A., Eisenacher M. (2022). The PRIDE database resources in 2022: A hub for mass spectrometry-based proteomics evidences. Nucleic Acids Res..

[27] McCarthy F.M., Wang N., Magee G.B., Nanduri B., Lawrence M.L., Camon E.B., Barrell D.G., Hill D.P., Dolan M.E., Williams W.P. (2006). AgBase: A functional genomics resource for agriculture. BMC Genom..

[28] Fonseka P., Pathan M., Chitti S.V., Kang T., Mathivanan S. (2021). FunRich enables enrichment analysis of OMICs datasets. J. Mol. Biol..

[29] Kanehisa M., Sato Y., Morishima K. (2016). BlastKOALA and GhostKOALA: KEGG Tools for Functional Characterization of Genome and Metagenome Sequences. J. Mol. Biol..

[30] Aramaki T., Blanc-Mathieu R., Endo H., Ohkubo K., Kanehisa M., Goto S., Ogata H. (2020). KofamKOALA: KEGG Ortholog assignment based on profile HMM and adaptive score threshold. Bioinformatics.

[31] Kanehisa M., Furumichi M., Sato Y., Kawashima M., Ishiguro-Watanabe M. (2023). KEGG for taxonomy-based analysis of pathways and genomes. Nucleic Acids Res..

[32] Lu S., Wang J., Chitsaz F., Derbyshire M.K., Geer R.C., Gonzales N.R., Gwadz M., Hurwitz D.I., Marchler G.H., Song J.S. (2020). CDD/SPARCLE: The conserved domain database in 2020. Nucleic Acids Res..

[33] Hallgren J., Tsirigos K., Pedersen M.D., Almagro Armenteros J.J., Marcatili P., Nielsen H., Krogh A., Winther O. (2022). DeepTMHMM predicts alpha and beta transmembrane proteins using deep neural networks. bioRxiv.

[34] Gíslason M.H., Nielsen H., Almagro Armenteros J.J., Johansen A.R. (2021). Prediction of GPI-anchored proteins with pointer neural networks. Curr. Res. Biotechnol..

[35] Xie Y., Zheng Y., Li H., Luo X., He Z., Cao S., Shi Y., Zhao Q., Xue Y., Zuo Z. (2016). GPS-Lipid: A robust tool for the prediction of multiple lipid modification sites. Sci. Rep..

[36] Zhao L., Poschmann G., Waldera-Lupa D., Rafiee N., Kollmann M., Stuhler K. (2019). OutCyte: A novel tool for predicting unconventional protein secretion. Sci. Rep..

[37] Almagro Armenteros J.J., Sønderby C.K., Sønderby S.K., Nielsen H., Winther O. (2017). DeepLoc: Prediction of protein subcellular localization using deep learning. Bioinformatics.

[38] Sperschneider J., Dodds P.N. (2022). EffectorP 3.0: Prediction of apoplastic and cytoplasmic effectors in fungi and oomycetes. Mol. Plant Microbe Interact..

[39] Silva B.M., Prados-Rosales R., Espadas-Moreno J., Wolf J.M., Luque-Garcia J.L., Goncalves T., Casadevall A. (2014). Characterization of *Alternaria infectoria* extracellular vesicles. Med. Mycol..

[40] Reis F.C.G., Gimenez B., Jozefowicz L.J., Castelli R.F., Martins S.T., Alves L.R., Oliveira H.C.d., Rodrigues M.L. (2021). Analysis of Cryptococcal Extracellular Vesicles: Experimental Approaches for Studying Their Diversity Among Multiple Isolates, Kinetics of Production, Methods of Separation, and Detection in Cultures of Titan Cells. Microbiol. Spectr..

[41] Cleare L.G., Zamith D., Heyman H.M., Couvillion S.P., Nimrichter L., Rodrigues M.L., Nakayasu E.S., Nosanchuk J.D. (2020). Media matters! Alterations in the loading and release of *Histoplasma capsulatum* extracellular vesicles in response to different nutritional milieus. Cell Microbiol..

[42] Thery C., Witwer K.W., Aikawa E., Alcaraz M.J., Anderson J.D., Andriantsitohaina R., Antoniou A., Arab T., Archer F., Atkin-Smith G.K. (2018). Minimal information for studies of extracellular vesicles 2018 (MISEV2018): A position statement of the International Society for Extracellular Vesicles and update of the MISEV2014 guidelines. J. Extracell. Vesicles.

[43] Dawson C.S., Garcia-Ceron D., Rajapaksha H., Faou P., Bleackley M.R., Anderson M.A. (2020). Protein markers for *Candida albicans* EVs include claudin-like Sur7 family proteins. J. Extracell. Vesicles.

[44] Garcia-Ceron D., Dawson C.S., Faou P., Bleackley M.R., Anderson M.A. (2021). Size-exclusion chromatography allows the isolation of EVs from the filamentous fungal plant pathogen *Fusarium oxysporum* f. sp. *vasinfectum* (Fov). Proteomics.

[45] Cohen M.J., Chirico W.J., Lipke P.N. (2020). Through the back door: Unconventional protein secretion. Cell Surf..

[46] Parreira V.d.S.C., Santos L.G.C., Rodrigues M.L., Passetti F. (2021). ExVe: The knowledge base of orthologous proteins identified in fungal extracellular vesicles. Comput. Struct. Biotechnol. J..

[47] Kalra H., Simpson R.J., Ji H., Aikawa E., Altevogt P., Askenase P., Bond V.C., Borras F.E., Breakefield X., Budnik V. (2012). Vesiclepedia: A compendium for extracellular vesicles with continuous community annotation. PLoS Biol..

[48] ten Have A., Espino J.J., Dekkers E., Van Sluyter S.C., Brito N., Kay J., Gonzalez C., van Kan J.A. (2010). The *Botrytis cinerea* aspartic proteinase family. Fungal Genet. Biol..

[49] Gee H.Y., Kim J., Lee M.G. (2018). Unconventional secretion of transmembrane proteins. Semin. Cell Dev. Biol..

[50] Gow N.A.R., Lenardon M.D. (2022). Architecture of the dynamic fungal cell wall. Nat. Rev. Microbiol..

[51] Choquer M., Rascle C., Gonçalves I.R., de Vallée A., Ribot C., Loisel E., Smilevski P., Ferria J., Savadogo M., Souibgui E. (2021). The infection cushion of *Botrytis cinerea*: A fungal ‘weapon’ of plant-biomass destruction. Environ. Microbiol..

[52] Frias M., Brito N., Gonzalez M., Gonzalez C. (2014). The phytotoxic activity of the cerato-platanin BcSpl1 resides in a two-peptide motif on the protein surface. Mol. Plant Pathol..

[53] Frias M., Gonzalez C., Brito N. (2011). BcSpl1, a cerato-platanin family protein, contributes to *Botrytis cinerea* virulence and elicits the hypersensitive response in the host. New Phytol..

[54] Zhao K., Bleackley M., Chisanga D., Gangoda L., Fonseka P., Liem M., Kalra H., Al Saffar H., Keerthikumar S., Ang C.S. (2019). Extracellular vesicles secreted by *Saccharomyces cerevisiae* are involved in cell wall remodelling. Commun. Biol..

[55] Mudholkar K., Fitzke E., Prinz C., Mayer M.P., Rospert S. (2017). The Hsp70 homolog Ssb affects ribosome biogenesis via the TORC1-Sch9 signaling pathway. Nat. Commun..

[56] Zhang H., Li Y., Dickman M.B., Wang Z. (2019). Cytoprotective Co-chaperone BcBAG1 Is a Component for Fungal Development, Virulence, and Unfolded Protein Response (UPR) of *Botrytis cinerea*. Front. Microbiol..

[57] Du W., Zhai P., Liu S., Zhang Y., Lu L. (2021). The Copper Chaperone CcsA, Coupled with Superoxide Dismutase SodA, Mediates the Oxidative Stress Response in *Aspergillus fumigatus*. Appl. Environ. Microbiol..

[58] Gleason J.E., Li C.X., Odeh H.M., Culotta V.C. (2014). Species-specific activation of Cu/Zn SOD by its CCS copper chaperone in the pathogenic yeast *Candida albicans*. J. Biol. Inorg. Chem..

[59] Cotoras M., Castro P., Vivanco H., Melo R., Mendoza L. (2013). Farnesol induces apoptosis-like phenotype in the phytopathogenic fungus Botrytis cinerea. Mycologia.

[60] Gioti A., Simon A., Le Pecheur P., Giraud C., Pradier J.M., Viaud M., Levis C. (2006). Expression profiling of *Botrytis cinerea* genes identifies three patterns of up-regulation in planta and an FKBP12 protein affecting pathogenicity. J. Mol. Biol..

[61] Wang J., Wang H., Zhang C., Wu T., Ma Z., Chen Y. (2019). Phospholipid homeostasis plays an important role in fungal development, fungicide resistance and virulence in *Fusarium graminearum*. Phytopathol. Res..

[62] Ma L., Salas O., Bowler K., Oren-Young L., Bar-Peled M., Sharon A. (2017). Genetic alteration of UDP-rhamnose metabolism in *Botrytis cinerea* leads to the accumulation of UDP-KDG that adversely affects development and pathogenicity. Mol. Plant Pathol..

[63] Santhanam P., Boshoven J.C., Salas O., Bowler K., Islam M.T., Saber M.K., van den Berg G.C., Bar-Peled M., Thomma B.P. (2017). Rhamnose synthase activity is required for pathogenicity of the vascular wilt fungus *Verticillium dahliae*. Mol. Plant Pathol..

[64] Kars I., Krooshof G.H., Wagemakers L., Joosten R., Benen J.A., van Kan J.A. (2005). Necrotizing activity of five *Botrytis cinerea* endopolygalacturonases produced in *Pichia pastoris*. Plant J..

[65] Zhang L., van Kan J.A. (2013). *Botrytis cinerea* mutants deficient in D-galacturonic acid catabolism have a perturbed virulence on *Nicotiana benthamiana* and Arabidopsis, but not on tomato. Mol. Plant Pathol..

[66] Zhang L., Hua C., Stassen J.H.M., Chatterjee S., Cornelissen M., van Kan J.A.L. (2014). Genome-wide analysis of pectate-induced gene expression in *Botrytis cinerea*: Identification and functional analysis of putative d-galacturonate transporters. Fungal Genet. Biol..

[67] Zhang L., Thiewes H., van Kan J.A. (2011). The D-galacturonic acid catabolic pathway in *Botrytis cinerea*. Fungal Genet. Biol..

[68] Beccaccioli M., Reverberi M., Scala V. (2019). Fungal lipids: Biosynthesis and signalling during plant-pathogen interaction. Front. Biosci..

[69] Chen D., Shu D., Wei Z., Luo D., Yang J., Li Z., Tan H. (2022). Combined transcriptome and proteome analysis of Bcfrp1 involved in regulating the biosynthesis of abscisic acid and growth in *Botrytis cinerea* TB-31. Front. Microbiol..

[70] Yan X., Que Y., Wang H., Wang C., Li Y., Yue X., Ma Z., Talbot N.J., Wang Z. (2013). The MET13 methylenetetrahydrofolate reductase gene is essential for infection-related morphogenesis in the rice blast fungus *Magnaporthe oryzae*. PLoS ONE.

[71] Sprenger M., Hartung T.S., Allert S., Wisgott S., Niemiec M.J., Graf K., Jacobsen I.D., Kasper L., Hube B. (2020). Fungal biotin homeostasis is essential for immune evasion after macrophage phagocytosis and virulence. Cell Microbiol..

[72] Siegmund U., Viefhues A., Fillinger S., Elad Y. (2016). Reactive Oxygen Species in the Botrytis—Host Interaction. Botrytis—The Fungus, the Pathogen and Its Management in Agricultural Systems.

[73] Zou X., Wei Y., Jiang S., Xu F., Wang H., Zhan P., Shao X. (2022). ROS Stress and Cell Membrane Disruption are the Main Antifungal Mechanisms of 2-Phenylethanol against *Botrytis cinerea*. J. Agric. Food Chem..

[74] Viefhues A., Heller J., Temme N., Tudzynski P. (2014). Redox systems in *Botrytis cinerea*: Impact on development and virulence. Mol. Plant Microbe Interact..

[75] Lopez-Cruz J., Oscar C.S., Emma F.C., Pilar G.A., Carmen G.B. (2017). Absence of Cu-Zn superoxide dismutase BCSOD1 reduces *Botrytis cinerea* virulence in Arabidopsis and tomato plants, revealing interplay among reactive oxygen species, callose and signalling pathways. Mol. Plant Pathol..

[76] Dautt-Castro M., Rosendo-Vargas M., Casas-Flores S. (2021). The Small GTPases in Fungal Signaling Conservation and Function. Cells.

[77] Zhang X., Jia X., Tian S., Zhang C., Lu Z., Chen Y., Chen F., Li Z., Su X., Han X. (2018). Role of the small GTPase Rho1 in cell wall integrity, stress response, and pathogenesis of *Aspergillus fumigatus*. Fungal Genet. Biol..

[78] An B., Li B., Qin G., Tian S. (2015). Function of small GTPase Rho3 in regulating growth, conidiation and virulence of *Botrytis cinerea*. Fungal Genet. Biol..

[79] Cox R., Mason-Gamer R.J., Jackson C.L., Segev N. (2004). Phylogenetic analysis of Sec7-domain-containing Arf nucleotide exchangers. Mol. Biol. Cell.

[80] Araiza-Cervantes C.A., Valle-Maldonado M.I., Patiño-Medina J.A., Alejandre-Castañeda V., Guzmán-Pérez J.B., Ramírez-Díaz M.I., Macias-Sánchez K.L., López-Berges M.S., Meza-Carmen V. (2021). Transcript pattern analysis of Arf-family genes in the phytopathogen *Fusarium oxysporum* f. sp. *lycopersici* reveals the role of Arl3 in the virulence. Antonie Van Leeuwenhoek.

