# Supplementary material for: The Adaptation of Botrytis cinerea Extracellular Vesicles Proteome to Surrounding Conditions: Revealing New Tools for Its Infection Process
Source: J Fungi (Basel). 2023 Aug 24;9(9):872. doi: 10.3390/jof9090872 (PMC10532283; doi:10.3390/jof9090872)
Supplement: Supplementary file 1 [file jof-09-00872-s001.zip › Supplementary Figures.pdf]

Supplementary data

The adaptation of *Botrytis cinerea* extracellular vesicles proteome to the surrounding conditions: Unrevealing new tools for its infection process.

Almudena Escobar-Niño[1]*, Anne Harzen[2], Sara C. Stolze [2], Hirofumi Nakagami[2, 3], Francisco J. Fernández-Acero[1,]

[1] Microbiology Laboratory, Institute for Viticulture and Agri-Food Research (IVAGRO), Faculty of Environmental and Marine Sciences. Department of Biomedicine, Biotechnology and Public Health, University of Cádiz, Puerto Real, Spain.

[2] Protein Mass Spectrometry, Max Planck Institute for Plant Breeding Research, Cologne, Germany.

[3] Basic Immune System of Plants, Max Planck Institute for Plant Breeding Research, Cologne, Germany.

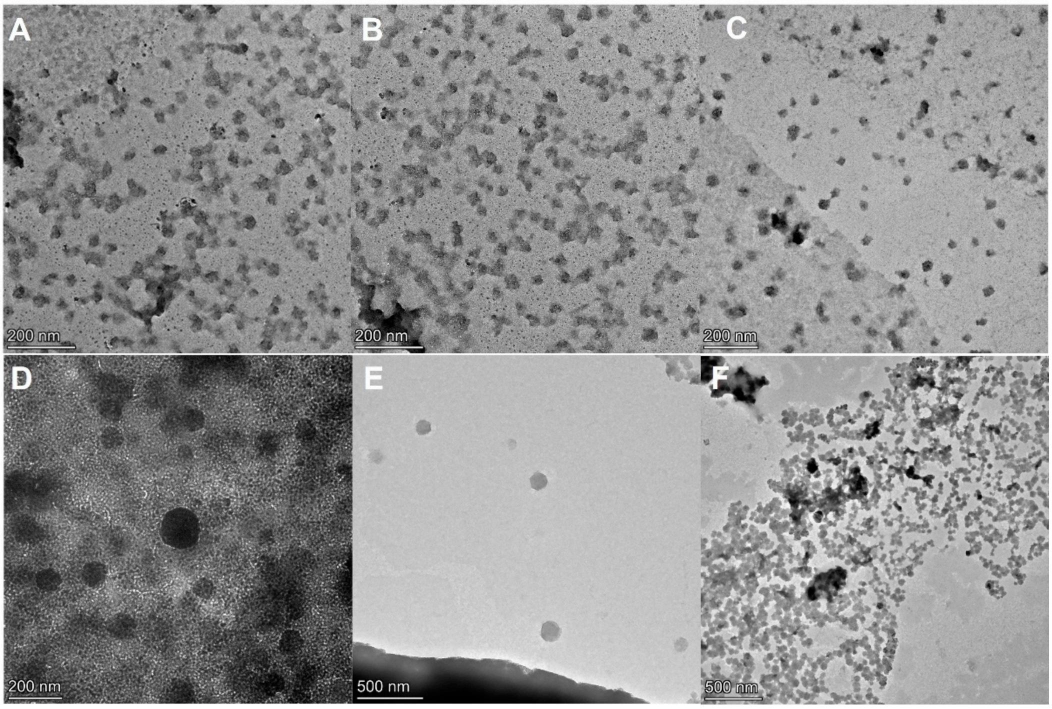

Supplementary Figure S1. ***B. cinerea* EVs at lower magnification (200nm-500nm).** TEM of EVs obtained from *B. cinerea* grown for 5 days in MSM liquid medium supplemented with TCW (A, B and C) or GLU (D, E and F).

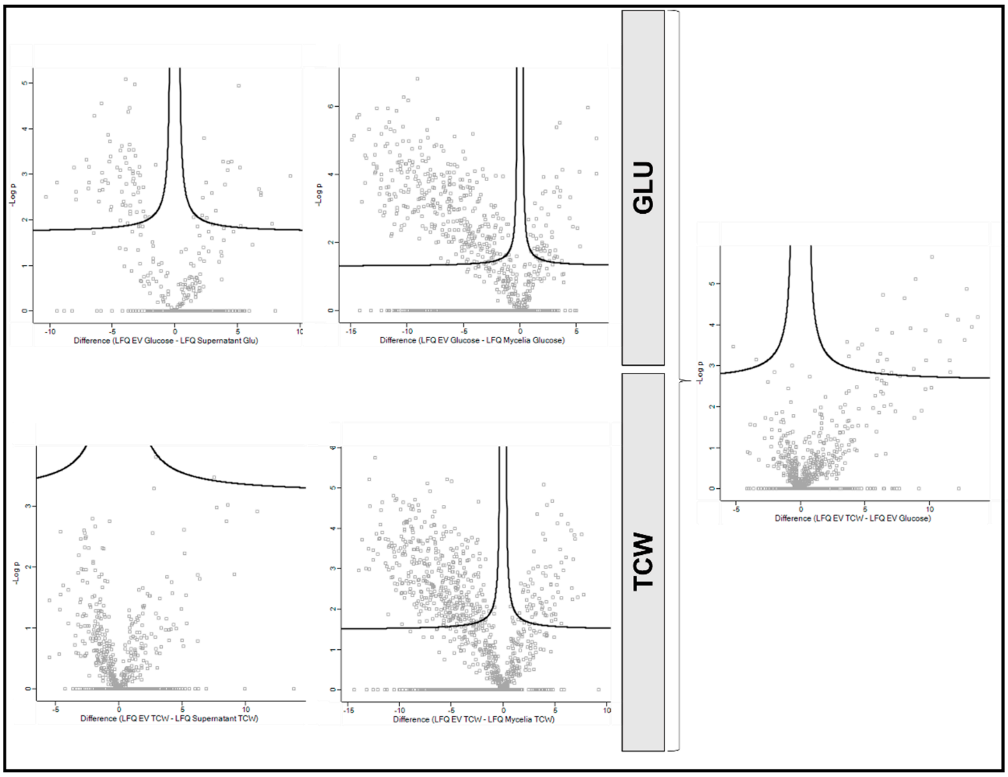

Supplementary Figure S2. Analysis on total protein distribution. Volcano plots of t-test results among sample classes using the list of 3442 proteins identified after filtration. Volcano plots were performed with Perseo 2.0.3.0. using permutation-based FDR calculation, FDR:0.01 and s:0.1. Note that there were not overrepresented proteins in EV TCW compared to Supernatant TCW, so exclusive and overrepresented proteins in EVs TCW and Supernatant TCW were just exclusive proteins.

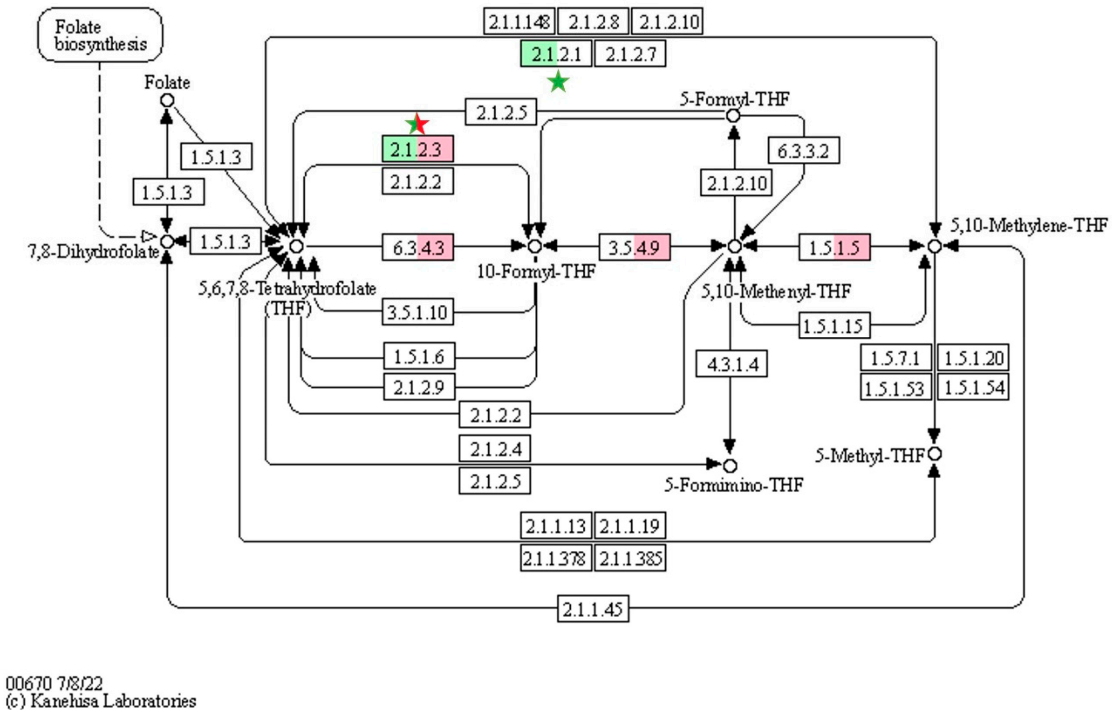

Supplementary Fig S3. Common KEGG pathway of EVs GLU and EVs TCW “One carbon pool by folate”. KO annotation of EVs TCW and EVs GLU relevant proteomes were used in Reconstruct KEGG mapper tool. Map ko00670 “One carbon pool by folate”. Red and Green: under TCW and GLU; Red: Under TCW; Green: under GLU; star: identified as exclusive or overexpressed protein in EVs proteome versus supernatant of this condition.

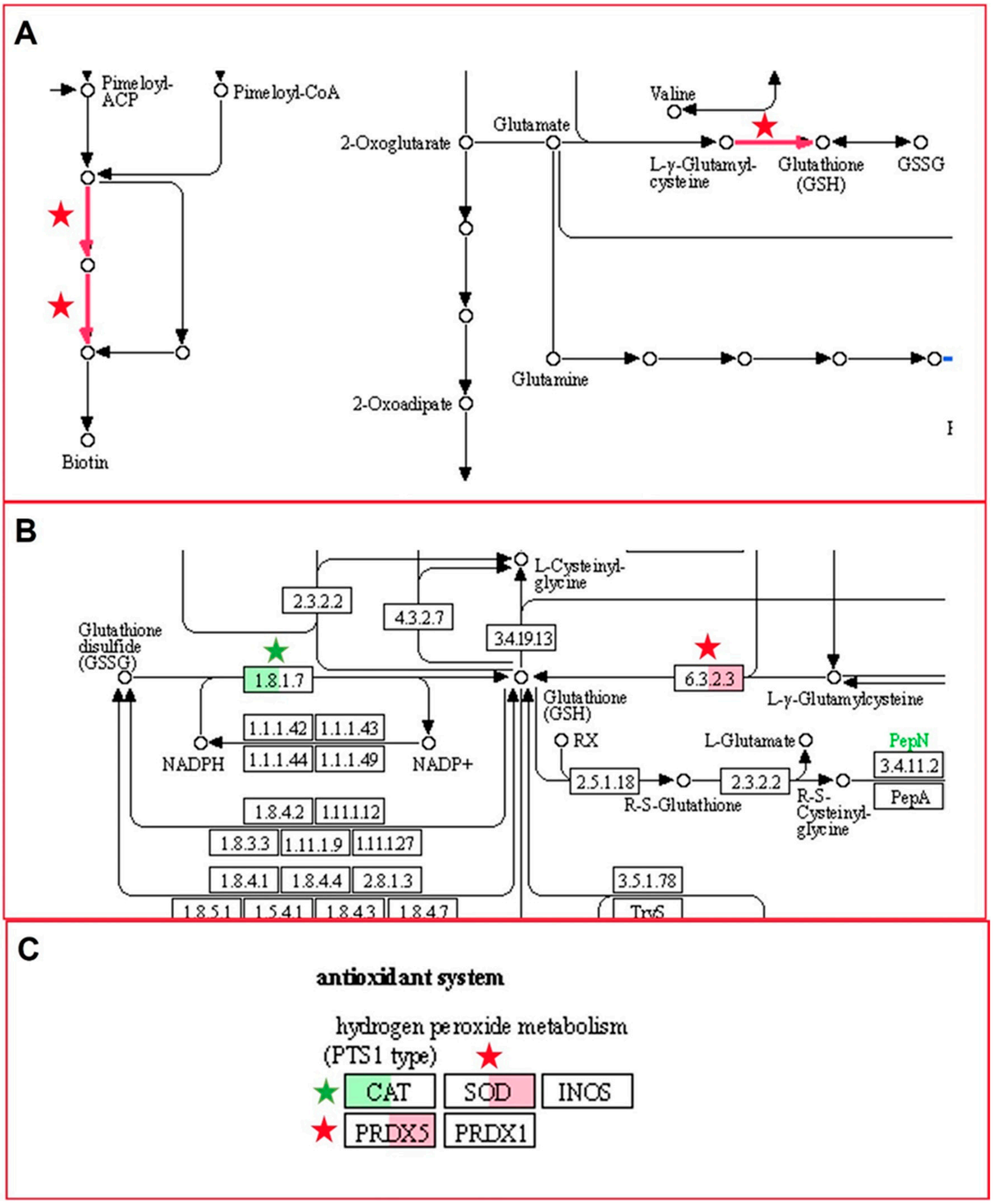

Supplementary Fig S4. Common KEGG pathway of EVs GLU and EVs TCW "Biosynthesis of cofactors" and "antioxidant system". (A) Detailed of biotin and glutathione biosynthesis from map01240 "Biosynthesis of cofactors"; (B) detail of glutathione generation from map00480 "Glutathione metabolism"; and (C) detail of antioxidant system from map04146 "peroxisome".
